# Supplemental N-acyl homoserine lactonase alleviates intestinal disruption and improves gut microbiota in broilers challenged by *Salmonella* Typhimurium

**DOI:** 10.1186/s40104-022-00801-4

**Published:** 2023-01-09

**Authors:** Weiwei Wang, Jingseng Ou, Hui Ye, Qingyun Cao, Changming Zhang, Zemin Dong, Dingyuan Feng, Jianjun Zuo

**Affiliations:** Guangdong Provincial Key Laboratory of Animal Nutrition Control, College of Animal Science, South China Agricultural University, Guangzhou, 510642 People’s Republic of China

**Keywords:** Broiler, Growth performance, Gut microbiota, Intestinal inflammation, N-acyl homoserine lactonase, Quorum quenching, *Salmonella* Typhimurium

## Abstract

**Background:**

*Salmonella* Typhimurium challenge causes a huge detriment to chicken production. N-acyl homoserine lactonase (AHLase), a quorum quenching enzyme, potentially inhibits the growth and virulence of Gram-negative bacteria. However, it is unknown whether AHLase can protect chickens against *S.* Typhimurium challenge. This study aimed to evaluate the effects of AHLase on growth performance and intestinal health in broilers challenged by *S.* Typhimurium. A total of 240 one-day-old female crossbred broilers (817C) were randomly divided into 5 groups (6 replicates/group): negative control (NC), positive control (PC), and PC group supplemented with 5, 10 or 20 U/g AHLase. All birds except those in NC were challenged with *S.* Typhimurium from 7 to 9 days of age. All parameters related to growth and intestinal health were determined on d 10 and 14.

**Results:**

The reductions (*P* < 0.05) in body weight (BW) and average daily gain (ADG) in challenged birds were alleviated by AHLase addition especially at 10 U/g. Thus, samples from NC, PC and PC plus 10 U/g AHLase group were selected for further analysis. *S.* Typhimurium challenge impaired (*P* < 0.05) intestinal morphology, elevated (*P* < 0.05) ileal inflammatory cytokines (*IL-1β* and *IL-8*) expression, and increased (*P* < 0.05) serum diamine oxidase (DAO) activity on d 10. However, AHLase addition normalized these changes. Gut microbiota analysis on d 10 showed that AHLase reversed the reductions (*P* < 0.05) in several beneficial bacteria (e.g. Bacilli, Bacillales and Lactobacillales), along with increases (*P* < 0.05) in certain harmful bacteria (e.g. Proteobacteria, Gammaproteobacteria, Enterobacteriaceae and *Escherichia*/*Shigella*) in PC group. Furthermore, AHLase-induced increased beneficial bacteria and decreased harmful bacteria were basically negatively correlated (*P* < 0.05) with the reductions of ileal *IL-1β* and *IL-8* expression and serum DAO activity, but positively correlated (*P* < 0.05) with the increased BW and ADG. Functional prediction revealed that AHLase abolished *S.* Typhimurium*-*induced upregulations (*P* < 0.05) of certain pathogenicity-related pathways such as lipopolysaccharide biosynthesis, shigellosis, bacterial invasion of epithelial cells and pathogenic *Escherichia coli* infection of gut microbiota.

**Conclusions:**

Supplemental AHLase attenuated *S.* Typhimurium-induced growth retardation and intestinal disruption in broilers, which could be associated with the observed recovery of gut microbiota dysbiosis.

**Supplementary Information:**

The online version contains supplementary material available at 10.1186/s40104-022-00801-4.

## Introduction

As one of the most prevalent pathogens isolated from poultry, *Salmonella* Typhimurium challenge causes a large variety of intestinal disorders such as inflammation and barrier dysfunction as well as gut microbota disturbance, thus impairing growth performance of chickens [1, 2]. Apart from inducing serious economic losses for chicken production, *S.* Typhimurium often results in food-borne salmonellosis in humans via the food chain (e.g. contaminated poultry meat), therefore threatening global public health. In the past few decades, antibiotics were broadly used to control *Salmonella* invasion in animals. Because of the severity of considerable disadvantages such as increases in bacterial resistance and antibiotic residue in animal products, the usage of antibiotics in feed has been prohibited in many countries and regions, which results in a high demand for seeking strategies to restrain *S.* Typhimurium infection in chickens.

One of the sophisticated ways of bacterial infection is quorum sensing (QS), which controls bacterial pathogenicity and accounts for various infection-related phenotypes of host [3]. QS is an intercellular communication system favoring coordination of bacterium-bacterium interactions and associations with host environment [3]. In essence, QS can be defined as a kind of cell-to-cell signaling mechanisms driving bacteria to respond to the perceived changes [3]. It is known that QS is mediated by several chemical signals (namely autoinducers) produced by gut bacteria [4]. The autoinducers are accumulated over bacterial growth until a reach of threshold concentration perceived by the bacteria and then combined with their homologous receptors, thereby triggering the self-regulation (activation or inhibition) of specific gene expression of bacteria with a subsequent benefit on their metabolism and activities (e.g. sporulation, biofilm formation and virulence gene expression) [3, 4]. Among autoinducers, N-acyl-homoserine lactone (AHL) is a critical member responsible for mediating QS of Gram-negative bacteria, which governs bacterial communities to create comfortable living conditions by synchronizing the vital activities of each member [4]. In terms of *Salmonella* (a typical Gram-negative bacterium), it can perceive and bind to AHL in gut to enhance its motility, biofilm-forming ability and toxin secretion, through which *Salmonella* can fortify its infection ability and subsequently exacerbates intestinal injuries of host [5, 6].

There has been an emphasis on a promising potential to use QS signals (e.g. AHL) as molecular targets for controlling bacterial infection [7]. It is knownt that QS can be abolished by adopting quorum quenching-related measures such as moderating accumulation of autoinducers through enzymatic degradation [8]. In spite of the possible variations in the length and substitution of acyl chain, AHLs produced by different bacteria share the same homoserine lactone moiety [8]. Hence, intestinal AHLs are able to be inactivated by N-acyl-homoserine lactonase (AHLase), an enzyme being efficient in degrading AHLs by hydrolyzing the lactone bonds [8]. This may allow an inhibition of Gram-negative bacteria (e.g. *S.* Typhimurium) in response to AHLase treatment. Indeed, previous in vitro studies revealed a mitigatory effect of AHLase on the QS-dependent pathogenicity such as virulence factor secretion and biofilm formation of certain Gram-negative pathogens [9, 10]. In in vivo studies, AHLase was suggested to increase the expression of anti-infectious factors (e.g. Toll-like receptor 5 and inducible nitric oxide synthase) and anti-inflammatory cytokine IL-10 in zebrafish infected with *Aeromonas hydrophila* [11]. Similar findings were obtained in several other studies where AHLase treatment repressed *Pseudomonas aeruginosa* (a typical Gram-negative pathogen) infection and blocked its ability to induce inflammatory injuries and mortality of mice [12, 13]. As a result, AHLase treatment represents a promising candidate for use as an approach against *S.* Typhimurium infection in animals. Although there were studies regarding the positive effects of AHLase on growth, survival and intestinal absorption in aquatic animals [14–16], almost no study was available regarding the application of AHLase in poultry production. Accordingly, the present study was conducted to probe if AHLase addition could attenuate *S.* Typhimurium-induced detriments to immunity, intestinal health and growth performance of broilers, aiming to provide a new strategy to limit *Salmonella* infection in chicken production.

## Materials and methods

### Animals and experimental design

The experimental animal protocols for this study were approved by the Animal Care and Use Committee of the South China Agricultural University (No. SCAU20210612). A total of 240 one-day-old female 817C (a commercial crossbred breed in China) broilers were randomly divided into 5 groups with 6 replicates and 8 birds per replicate. The initial body weight was similar across all the replicates. The treatments were as follows: negative control (NC) (received a basal diet and free of challenge), positive control (PC) (received a basal diet and *S.* Typhimurium challenge), PC supplemented with 5, 10 or 20 U/g AHLase. AHLase product (the theoretical value of enzyme activity was 5000 U/g) was obtained from Beijing Challenge Group (China) and corn starch was used as the carrier. Thereby, AHLase product was supplemented to diet at the expense of corn powder. The addition amount of AHLase in diet and its preparation were based on the study of Cao et al. [15] and our preliminary experiment. The actual activity (4852 U/g) of AHLase in this supplement was determined using high performance liquid chromatography with N-(3-oxo-octanoyl)-L-homoserine lactone (3-oxo-C8-HSL) (Santa Cruz Biotech., Dallas, USA) used as the substrate [15]. Birds were raised in three-tier cages and exposed to 16 h of light per day. Room temperature was maintained at 34 °C during the first week and gradually reduced to 30 °C on d 14. All birds had free access to the mash diet and water. The composition of basal diet is shown in Table 1.

**Table 1:** Composition of the basal diet (air-dry basis)

Ingredients	Content, %
Corn	54.70
Soybean meal (CP 43.6%)	38.10
Soybean oil	2.60
Limestone	1.20
Dicalcium phosphate	2.00
Sodium chloride	0.26
Lysine	0.01
*DL*-Methionine (98%)	0.13
Premix^1^	1.00
Nutrient levels^2^	
Metabolizable energy, MJ/kg	12.55
Crude protein, %	21.10
Calcium, %	1.00
Available phosphorus, %	0.46
Lysine, %	1.2
Methionine, %	0.5

^1^Supplied per kilogram of diet: Cu, 9.5 mg; Zn, 60 mg; Fe, 70 mg; Mn, 121 mg; Se, 0.45 mg; I, 1.4 mg; vitamin A, 5000 IU; thiamin, 2.5 mg; riboflavin, 15 mg; pyridoxine, 4 mg; vitamin D, 80.75 mg; tocopherol, 31 mg; menadione, 1.6 mg; pantothenic acid, 60 mg; niacin, 15 mg; biotin, 0.5 mg; folic acid, 1.5 mg; choline, 450 mg

^2^Values represent analyzed levels of nutrients

### Oral challenge and sampling

The *S.* Typhimurium strain (ATCC14028, Food Microbial Safety Engineering Technology R&D Center of Guangdong Province, Guangzhou, China) was inoculated in *Salmonella-Shigella* agar and incubated at 37 °C for 12 h. The single colonies were selected and inoculated in lactose broth, followed by culture in incubator shaker (37 °C, 180 r/min) overnight. The bacteria were enumerated by plating on *Salmonella-Shigella* agar at 37 °C for 24 h. From 7 to 9 days of age, each bird in PC and AHLase groups was orally gavaged with 2 mL of *S.* Typhimurium culture (2 × 10^9^ CFU/mL) for three consecutive days, while NC birds received the same amount of lactose broth. At 10 and 14 days of age, birds were randomly selected from each replicate (6 birds/group), blood was taken from the wing vein and serum samples were obtained by centrifugation of blood at 3000 r/min for 10 min at 4 °C. Birds were then euthanized for separation of liver and spleen. Afterwards, the midpoints of jejunum and ileum of each bird were removed and cut into two segments, one of which was fixed in 4% paraformaldehyde solution, and the other one was froze in liquid nitrogen and stored at −80 °C. Besides, cecal chyme was harvested from each bird on d 10 for gut microbiota analysis.

### Determination of growth performance, relative organ weight and serum parameter

Body weight was recorded for each replicate on d 10 and 14. Average daily gain (ADG) during 1–10 d and 1–14 d was then calculated. The collected liver and spleen were weighed for the measurement of relative organ weight, as calculated by the ratio of organ weight (g) to live body weight (kg) × 100%. Serum diamine oxidase (DAO) activity was measured using a commercial kit (Solarbio Co. Ltd., Beijing, China) with a Multiskan SkyHigh microplate reader (Thermo Fisher Scientific, Waltham, USA).

### Measurement of intestinal morphology

The fixed jejunum and ileum samples were embedded in paraffin and stained by hematoxylin-eosin to obtain cross-sections. Regarding each section, the intact and representative villi were selected for measurement of intestinal morphology using a light microscope. Villus height (VH) was measured from the villous tip to the villus-crypt joint, while crypt depth (CD) was defined as the depth of invagination between adjacent villi. Villus height to crypt depth ratio (V/C) was then figured out.

### RNA extraction and real-time PCR

Total RNA from jejunum and ileum was extracted and purified using the FastPure Cell/Tissue Total RNA Isolation Kit V2 (Vazyme Biotech. Co. Ltd., Nanjing, China) following the manufacturer's protocols. The extracted RNA was dissolved in RNase-free water and quantified using a NanoDrop-2000 spectrophotometer (Thermo Fisher Scientific, Waltham, USA). RNA purity was evaluated by measuring the absorbance ratio at 260:280 nm, while RNA integrity was confirmed by detection of the 18S and 28S bands after electrophoresis in 1% agarose gels. RNA samples were then reverse transcribed into cDNA samples using the HiScript II qRT SuperMix for qPCR (Vazyme Biotech. Co. Ltd., Nanjing, China). Real-time PCR for measuring gene expression was performed using the 2×Taq Master Mix (Vazyme Biotech. Co. Ltd., Nanjing, China) in a CFX96Touch Real-Time PCR system (Bio-Rad Laboratories, Hercules, USA). Reduced glyceraldehyde-phosphate dehydrogenase (*GAPDH*) was used as the internal control. Primer sequences for *GAPDH* and the target genes including interlukin (*IL*)-*1β*, *IL*-*8*, tumor necrosis factor α (*TNF-α*), claudin-1, occludin and zonula occluden 1 (*ZO-1*) are shown in Table S1. The results of relative mRNA expression of genes were calculated using the 2^-ΔΔCt^ method [17].

### High-throughput absolute quantification sequencing of gut microbiota

The observed dynamics in routine 16S (relative quantification) sequencing of microbiota may be imprecise, since it can only obtain the relative abundance (RA)-related information disregarding of the potential difference in absolute abundances (AA) of bacteria [18]. In this study, gut microbiota was analyzed by the 16S sequencing approach integrated with the absolute quantitative PCR technique (denoted as high-throughput absolute quantification 16S sequencing) according to previous studies [19, 20]. Briefly, bacterial genomic DNA was firstly isolated from cecal chyme of broilers on d 10 using FastDNA™ SPIN Kit (MP Biomedicals, Irvine, CA, USA). The concentration and quality of isolated DNA were verified using gel electrophoresis and Qubit 3.0 fluorometer (Thermo Fisher Scientific, Waltham, USA). Thereafter, different spike-in sequences with different concentrations of internal standards were added to the sample DNA pools. The spike-in sequences contained conserved regions identical to those of natural 16S rRNA genes and artifical variable regions different from nucleotide sequences in the public databases, thus serving as internal standards and allowing the absolute quantification of bacterial taxa [19, 20]. The spike-in sequences together with bacterial 16S rDNA sequences of each sample spanning the variable regions V3-V4 using primers 341 F (5′-CCTACGGGNGGCWGCAG-3′) and 805 R (5′-GACTACHVGGGTATCTAATCC-3′) were amplified and then sequenced by the Genesky Biotechnologies Inc. (Shanghai, China) on Illumina Novaseq platform (Illumina, San Diego, USA).

The principal component analysis (PCA), principal coordinates analysis (PCoA) and non-metric multidimensional scaling (NMDS) were employed to evaluate pairwise distances among samples and to establish β-diversity. Linear discriminant analysis (LDA) combined effect size measurements (LEfSe) and Kruskal-Wallis rank sum test were used to detect bacterial differences among groups. Functional contents of gut metagenome were predicted using the Phylogenetic Investigation of Communities by Reconstruction of Unobserved State (PICRUSt). Spearman’s correlation analysis was performed for the correlations betweeen gut microbiota and other parameters.

### Statistical analysis

Data are presented as mean ± standard deviation and analyzed by one-way ANOVA using the general linear model procedure of SPSS 20.0. Differences among different groups were detected by Duncan’s multiple comparisons. Significance was set at *P* < 0.05. Kruskal-Wallis rank sum tests were used to detect differences in the abundances of bacterial members among groups.

## Results

### Growth performance

Broilers in PC group had lower (*P <* 0.05) BW on d 10 and 14 as well as ADG on d 1–10 and d 1–14 when compared with those in NC group (Table 2). However, these parameters except ADG on d 1–10 in 5 U/g AHLase group were greater (*P <* 0.05) than those in PC group by 4.92%, 4.94% and 6.45%, respectively. Remarkably, AHLase addition at 10 U/g increased (*P* < 0.05) all the above parameters by 6.47%, 6.80%, 8.97% and 8.95%, respectively, when compared with PC group. However, 20 U/g AHLase group displayed no improvements (*P* > 0.05) in broiler growth performance relative to PC group. Based on the above findings, AHLase addition at a dosage of 10 U/g was suitable for alleviating growth retardation of *S.* Typhimurium-challenged broilers. Thereby, samples from NC group, PC group and 10 U/g AHLase group (PC birds fed with 10 U/g AHLase) were selected for further analysis.

**Table 2:** Effect of N-acyl homoserine lactonase (AHLase) on growth performance^1^ of broilers challenged by *Salmonella* Typhimurium

	BW, g			ADG, g	
	Day 1	Day 10	Day 14	Days 1–10	Days 1–14
NC^2^	40.31±0.02	113.06±2.15^a^	180.21±3.12^a^	8.09±0.24^a^	10.76±0.24^a^
PC	40.22±0.02	105.64±0.42^b^	165.09±1.87^c^	7.36±0.10^b^	9.61±0.14^c^
5 U/g AHLase	40.26±0.03	110.84±2.41^a^	173.24±2.99^ab^	7.84±0.27^ab^	10.23±0.23^ab^
10 U/g AHLase	40.26±0.03	112.48±0.65^a^	176.32±1.58^ab^	8.02±0.07^a^	10.47±0.12^ab^
20 U/g AHLase	40.26±0.03	109.42±0.21^ab^	171.11±1.09^bc^	7.69±0.02^ab^	10.07±0.08^bc^
*P*-value	0.279	0.016	0.003	0.025	0.004

^a-c^ Values within a column with unlike superscript letters differ significantly (*P* < 0.05)

^1^ *BW* body weight, *ADG* average daily gain

^2^ *NC* negative control (broilers were free of challenge), *PC* positive control (broilers were challenged with *S.* Typhimurium from 7 to 9 days of age); *AHLase*, PC broilers supplemented with 10 U/g AHLase

### Relative organ weight and serum DAO activity

**Fig. 1:**
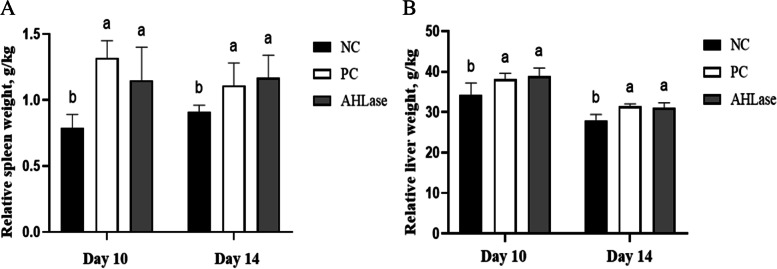
Effect of N-acyl homoserine lactonase (AHLase) on the relative organ weight of broilers challenged by *Salmonella* Typhimurium. ^a,b^ Values within a column with unlike superscript letters differ significantly (*P* < 0.05). NC, negative control (broilers were free of challenge); PC, positive control (broilers were challenged with *S.* Typhimurium); AHLase, PC broilers supplemented with 10 U/g AHLase

**Fig. 2:**
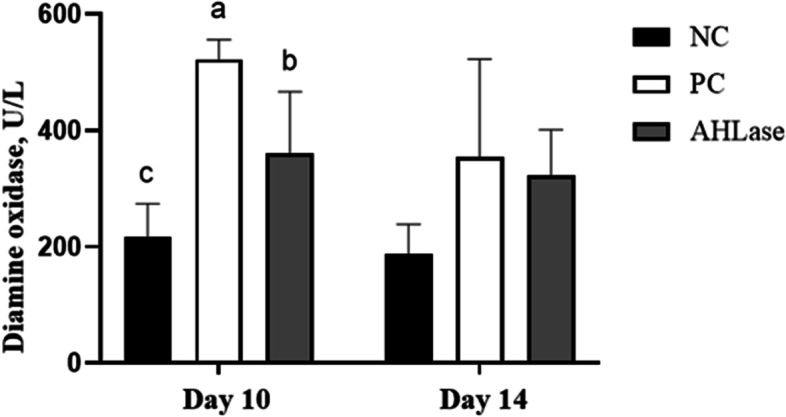
Effect of N-acyl homoserine lactonase (AHLase) on serum diamine oxidase activity of broilers challenged by *Salmonella* Typhimurium. ^a-c^ Values within a column with unlike superscript letters differ significantly (*P* < 0.05). NC, negative control (broilers were free of challenge); PC, positive control (broilers were challenged with *S.* Typhimurium); AHLase, PC broilers supplemented with 10 U/g AHLase

There were increases (*P <* 0.05) in the relative weights of liver and spleen of broilers on d 10 and 14 in PC group in comparison with NC group (Fig. 1). However, the relative weights of liver and spleen on d 10 and 14 did not differ (*P >* 0.05) between AHLase and PC groups. Regarding serum DAO activity, it was elevated (*P <* 0.05) in PC group on d 10 relative to NC group (Fig. 2). In comparison, serum DAO activity in AHLase group on d 10 was lower (*P <* 0.05) than that in PC group but still higher (*P <* 0.05) than that in NC group.

### Intestinal morphology

Broilers in PC group showed increases (*P <* 0.05) in both jejunal and ileal CD coupled with reductions (*P <* 0.05) in jejunal and ileal V/C on d 10 versus NC group (Table 3). Comparatively, there were reductions (*P <* 0.05) in jejunal and ileal CD, as well as elevations (*P <* 0.05) in ileal VH, jejunal and ileal V/C in AHLase group on d 10 compared with PC group. With respect to intestinal morphology of birds on d 14, we noted an increase (*P <* 0.05) in ileal CD along with reductions (*P <* 0.05) of jejunal and ileal V/C in PC group versus NC group, however, these parameters were all better (*P <* 0.05) in AHLase group compared with PC group.

**Table 3:** Effect of N-acyl homoserine lactonase (AHLase) on intestinal morphology^1^of broilers challenged by *Salmonella* Typhimurium

		Jejunum			Ileum		
		VH, μm	CD, μm	V/C	VH, μm	CD, μm	V/C
Day 10	NC^2^	626.22±62.64	106.85±10.07^b^	5.86±0.59^a^	485.79±20.67^b^	117.68±2.74^b^	4.13±0.18^b^
	PC	573.60±74.18	163.69±30.66^a^	3.83±0.13^b^	467.91±28.24^b^	153.65±30.09^a^	3.05±0.18^c^
	AHLase	634.42±20.21	109.20±9.54^b^	5.81±0.19^a^	547.93±15.57^a^	114.20±16.40^b^	4.80±0.14^a^
	*P*-value	0.352	0.002	< 0.001	0.002	0.037	< 0.001
Day 14	NC^2^	649.46±40.12	100.85±13.47	6.95±0.41^a^	562.59±48.41	90.03±2.67^b^	6.29±0.42^a^
	PC	606.08±126.04	108.85±13.54	5.41±0.28^b^	526.58±34.54	113.93±6.95^a^	5.01±0.52^b^
	AHLase	642.24±82.99	88.87±17.50	6.96±0.31^a^	548.53±13.88	86.64±8.64^b^	6.54±0.68^a^
	*P*-value	0.772	0.267	< 0.001	0.386	< 0.001	0.003

^a,b^ Values within a column with unlike superscript letters differ significantly (*P* < 0.05)

^1^ *VH* villus height, *CD* crypt depth, *V/C* villus height to crypt depth ratio

^2^ *NC* negative control (broilers were free of challenge), *PC* positive control (broilers were challenged with *S.* Typhimurium from 7 to 9 d of age); *AHLase*, PC broilers supplemented with 10 U/g AHLase

### Relative mRNA expression of intestinal genes

**Fig. 3:**
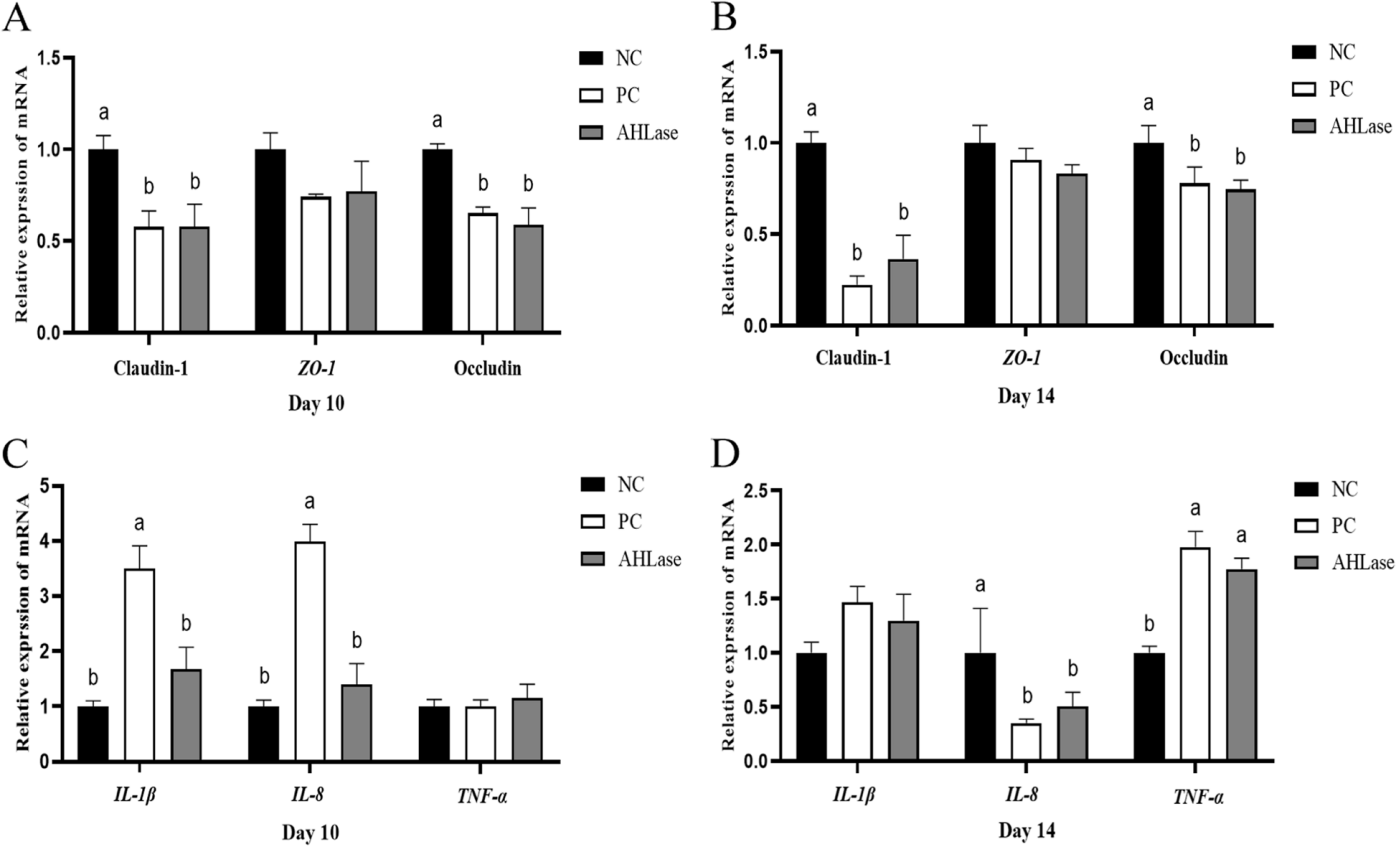
Effect of N-acyl homoserine lactonase (AHLase) on the relative mRNA expression of ileal tight junction proteins (**A**, **B**) and inflammatory cytokines (**C**, **D**) of broilers challenged by *Salmonella* Typhimurium. ^a,b^ Values with different superscripts differ significantly (*P* < 0.05). *ZO-1*, zonula occludens 1; *IL*, interleukin; *TNF-α*, tumor necrosis factor α. NC, negative control (broilers were free of challenge); PC, positive control (broilers were challenged with *S.* Typhimurium); AHLase, PC broilers supplemented with 10 U/g AHLase

Compared with NC group, PC group had a reduced (*P* < 0.05) expression of ileal Claudin-1 and Occludin on both d 10 and 14 (Fig. 3A, B). No difference (*P >* 0.05) was observed in the expression of ileal TJ proteins including Claudin-1, Occludin and *ZO-1* between PC and AHLase groups. Regarding ileal inflammatory cytokines, the expression of *IL-1β* and *IL-8* on d 10 was higher (*P* < 0.05) in PC group versus NC group (Fig. 3C**)**. Besides, PC group showed a reduction (*P* < 0.05) in ileal *IL-8* expression with an elevation (*P* < 0.05) of ileal *TNF-α* expression on d 14 as compared with NC group (Fig. 3D**)**. A reduction (*P <* 0.05) in ileal *IL-1β* and *IL-8* expression on d 10 rather than d 14 was observed in AHLase group relative to PC group.

### Gut microbiota

#### Diversity of gut microbiota

**Fig. 4:**
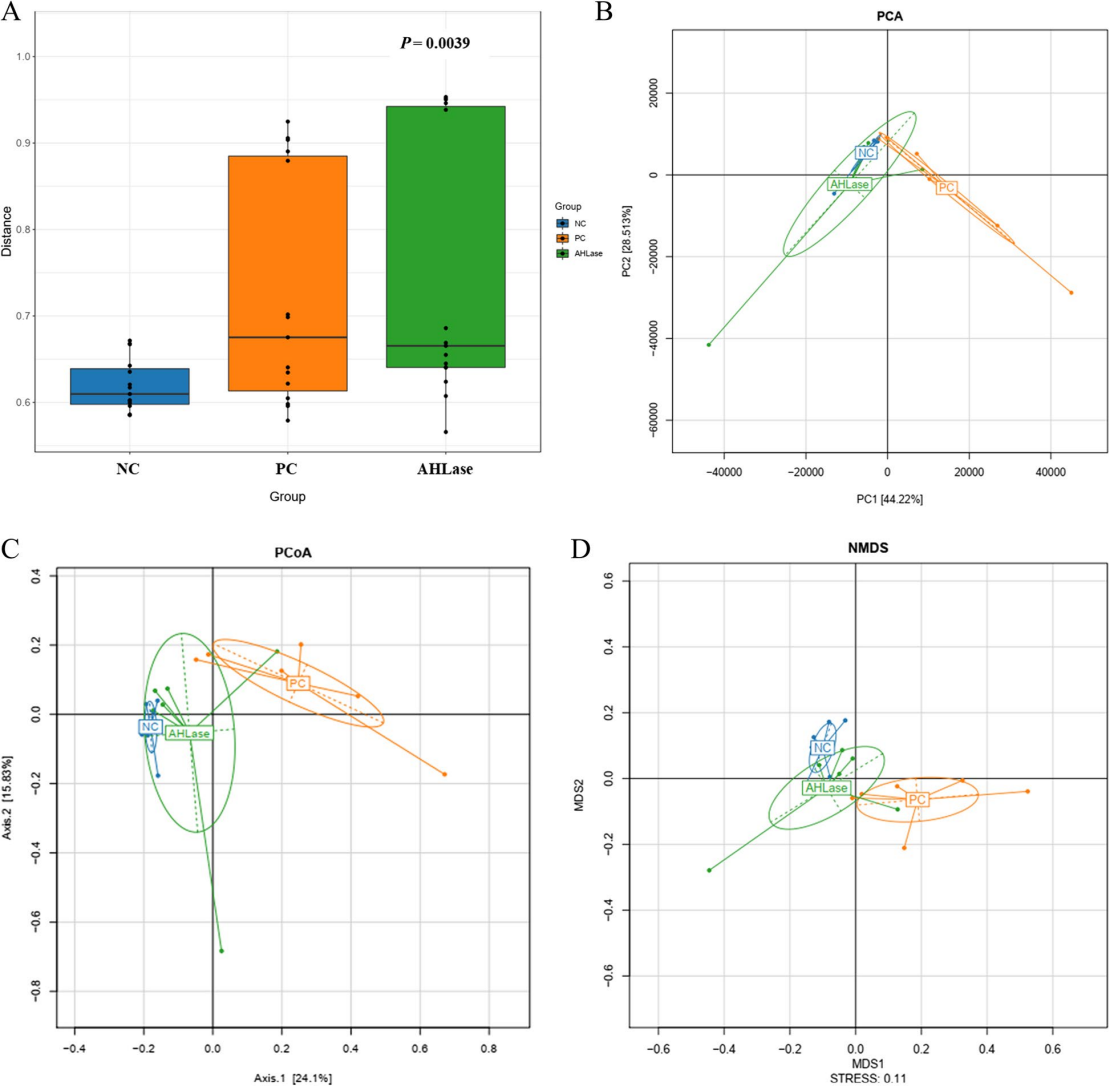
Beta-diversity analysis of gut microbiota of broilers on d 10. **A** ADONIS analysis (similarity analysis). **B** Non-metric multidimensional scaling (NMDS). **C** Principal component analysis (PCA). **D** Principal co-ordinates analysis (PCoA). NC, negative control (broilers were free of challenge); PC, positive control (broilers were challenged with *S.* Typhimurium); AHLase, PC broilers supplemented with 10 U/g AHLase

No difference (*P* > 0.05) occurred in the α-diversity indicators of gut microbiota among groups (Fig. S1). ADONIS analysis revealed a difference (*P* < 0.05) in the β-diversity (similarity) of gut microbiota among groups (Fig. 4A). This was visualized by both PCA, PCoA and NMDS plots, which defined groups where the samples from NC and PC groups occupied obviously distinct positions (Fig. 4B–D), however, only a little separation of microbiota was noted between PC and AHLase groups.

#### Composition of gut microbiota

**Fig. 5:**
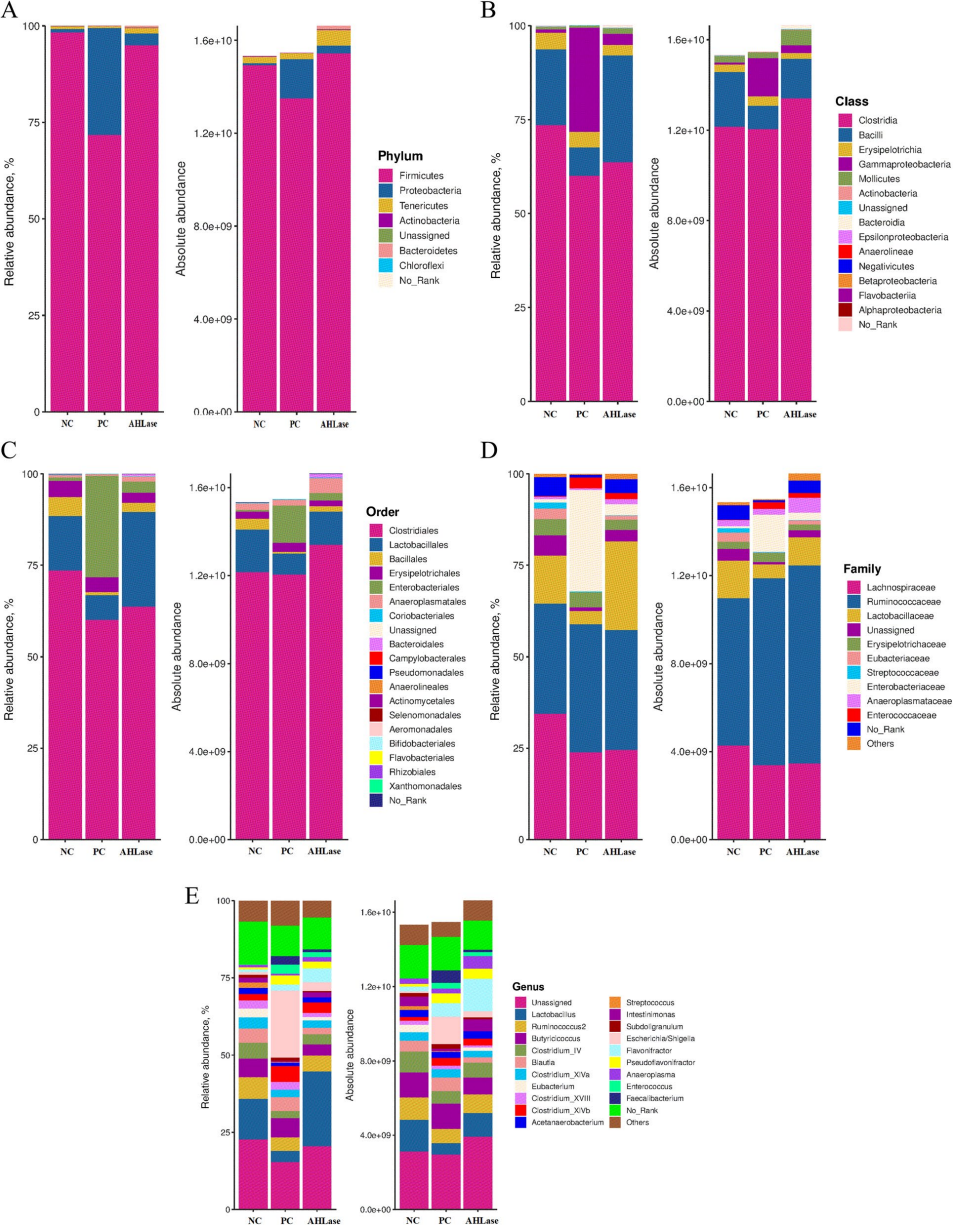
Gut microbial composition of broilers on d 10. NC, negative control (broilers were free of challenge); PC, positive control (broilers were challenged with *S.* Typhimurium); AHLase, PC broilers supplemented with 10 U/g AHLase

The main phylum of broiler gut microbiota was Firmicutes, followed by Proteobacteria (Fig. 5A). The RA of Firmicutes and Proteobacteria in AHLase group (94.95% and 3.06%) were higher than PC group (71.66% and 27.73%) and close to NC group (98.31% and less than 1%) (Fig. S2). Similar findings were obtained for the AA of Firmicutes and Proteobacteria among groups (Fig. 5A). Within Firmicutes, the majority belonged to the classes Clostridia and Bacilli (Fig. 5B). There were reductions in the RA or AA of Clostridia and Bacilli concurrent with increases in the RA and AA of Gammaproteobacteria in PC group versus NC group, while AHLase group showed increases in the RA and AA of Clostridia and Bacilli as well as reductions in Gammaproteobacteria as compared with PC group. Order level analysis manifested that the gut microbiota was dominated by Clostridiales and Lactobacillales, whose RA and AA were increased in AHLase group relative to PC group (Fig. 5C). At family level, the predominant members were Lachnospiraceae, Ruminococcaceae and Lactobacillaceae, whose RA and AA were distintly different among groups (Fig. 5D). The dominating genera in gut were the unassigned genus, *Lactobacillus* and *Ruminococcus* (Fig. 5E). Among the genera, the RA of *Escherichia*/*Shigella* was highest in PC group (21.66%), ranked by AHLase group (2.92%) and NC group (very little) (Fig. S2). Inversely, the RA of *Lactobacillus* was highest in AHLase group (24.25%), followed by NC group (13.11%) and PC group (3.65%). Similar patterns were found for the AA of *Lactobacillus* and *Escherichia*/*Shigella* among groups.

#### Differential members in gut microbiota among groups

**Fig. 6:**
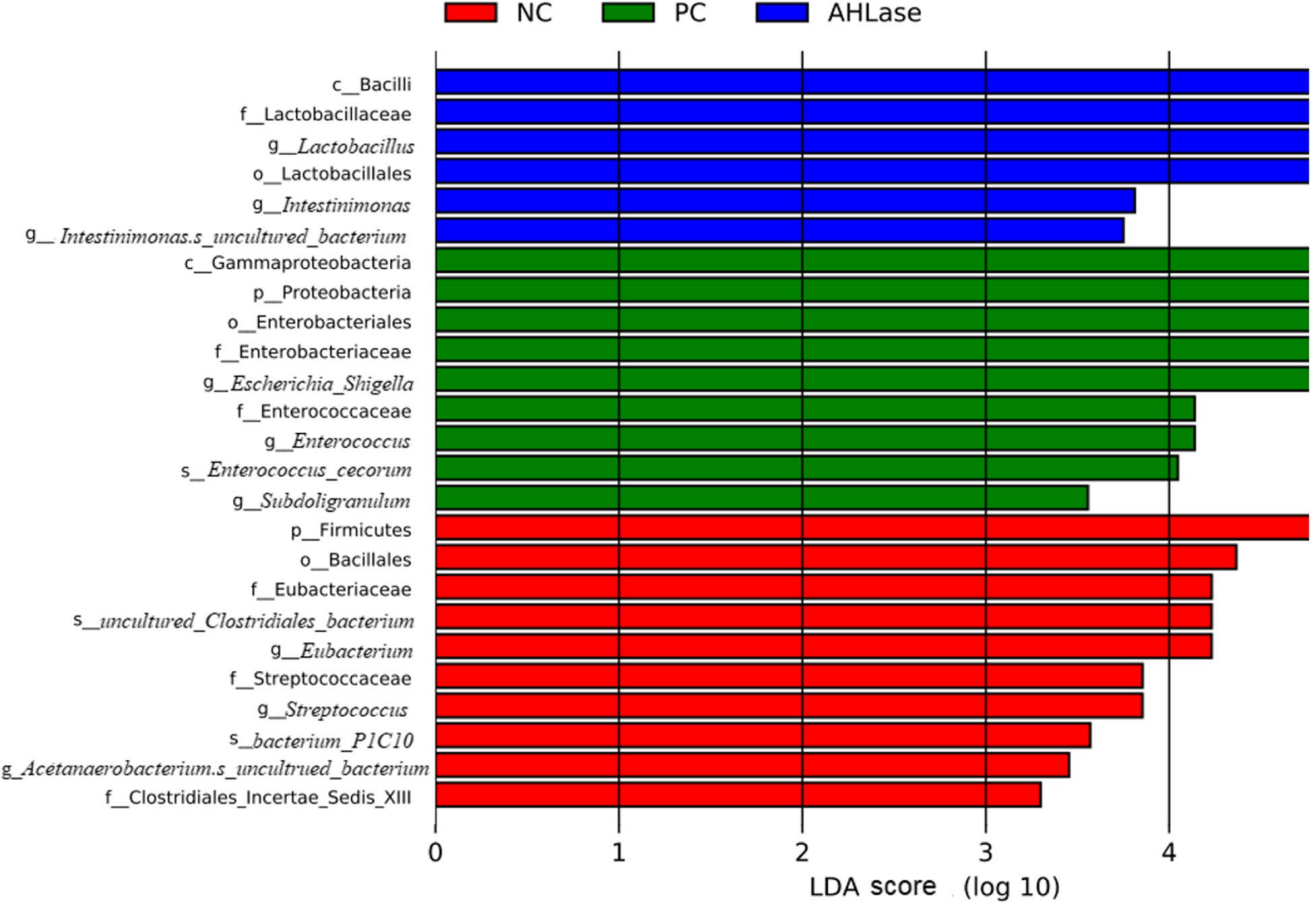
Linear discriminant analysis (LDA) combined effect size measurements (LEfSe) analysis of bacterial richness (*P* < 0.05, LDA > 3.0) in gut microbiota of broilers on d 10. NC, negative control (broilers were free of challenge); PC, positive control (broilers were challenged with *S.* Typhimurium); AHLase, PC broilers supplemented with 10 U/g AHLase

**Fig. 7:**
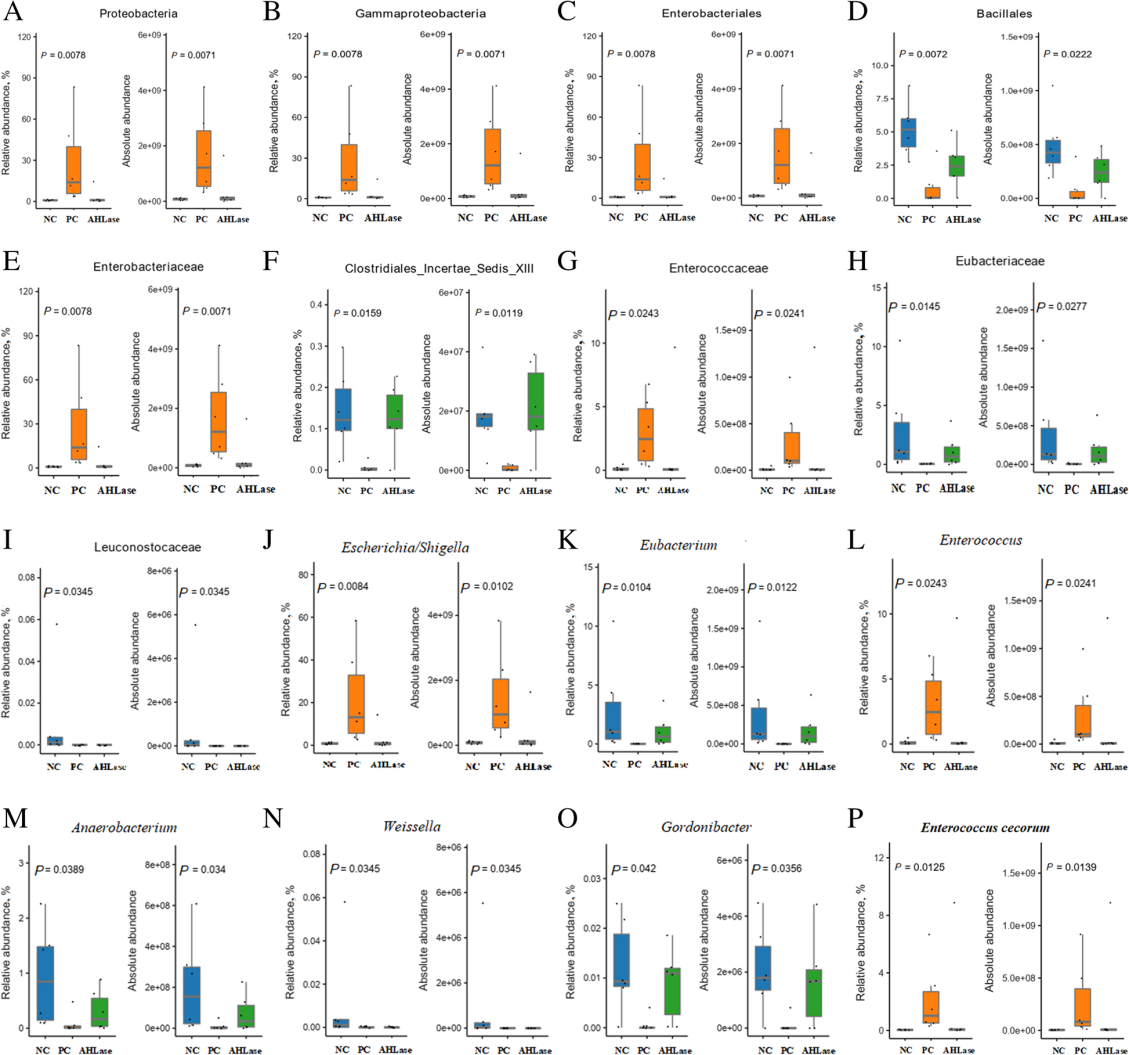
Bacterial members differ in both the relative and absolute abundances at different taxonomic levels (**A**, phylum; **B**, class; **C**~**D**, order; **E**~**I**, family; **J**~**O**, genus; **P**, species) of gut microbiota among groups on d 10. Differences were identified by Kruskal-Wallis analysis. NC, negative control (broilers were free of challenge); PC, positive control (broilers were challenged with *S.* Typhimurium); AHLase, PC broilers supplemented with 10 U/g AHLase

LEfSe analysis was used to identify the bacterial richness (*P* < 0.05, LDA > 3.0) in groups. As shown in Fig. 6**,** there were certain bacterial members such as phylum Firmicutes, order Bacillales and genus *Eubacterium* enriched in NC group. The phylum Proteobacteria, class Gammaproteobacteria, order Enterobacteriales, families Enterobacteriaceae and Enterococcaceae, genera *Escherichia*/*Shigella*, *Subdoligranulum* and *Enterococcus*, together with species *Enterococcus cecorum* were enriched in PC group. In comparison, AHLase group was differentially enriched with Bacilli, Lactobacillales, Lactobacillaceae, *Lactobacillus* and *Intestinimonas*. Species differences were further detected by Kruskal-Wallis analysis (Fig. 7), which showed that both the RA and AA of potentially harmful bacteria including Proteobacteria, Gammaproteobacteria, Enterobacteriales, Enterobacteriaceae, Enterococcaceae, *Escherichia*/*Shigella*, *Enterococcus* and *E. cecorum* in AHLase group were lower (*P* < 0.05) than PC group and close to NC group. In contrast, PC group had reduced (*P* < 0.05) RA and AA of Bacillales, Clostridiales_Incertae_Sedis_XIII, Eubacteriaceae, Leuconostocaceae, *Eubacterium*, *Anaerobacterium*, *Weissella* and *Gordonibacte* versus NC group, however, both the RA and AA of most of these bacteria were comparable between AHLase and NC groups.

#### Functional prediction of gut microbiota

**Fig. 8:**
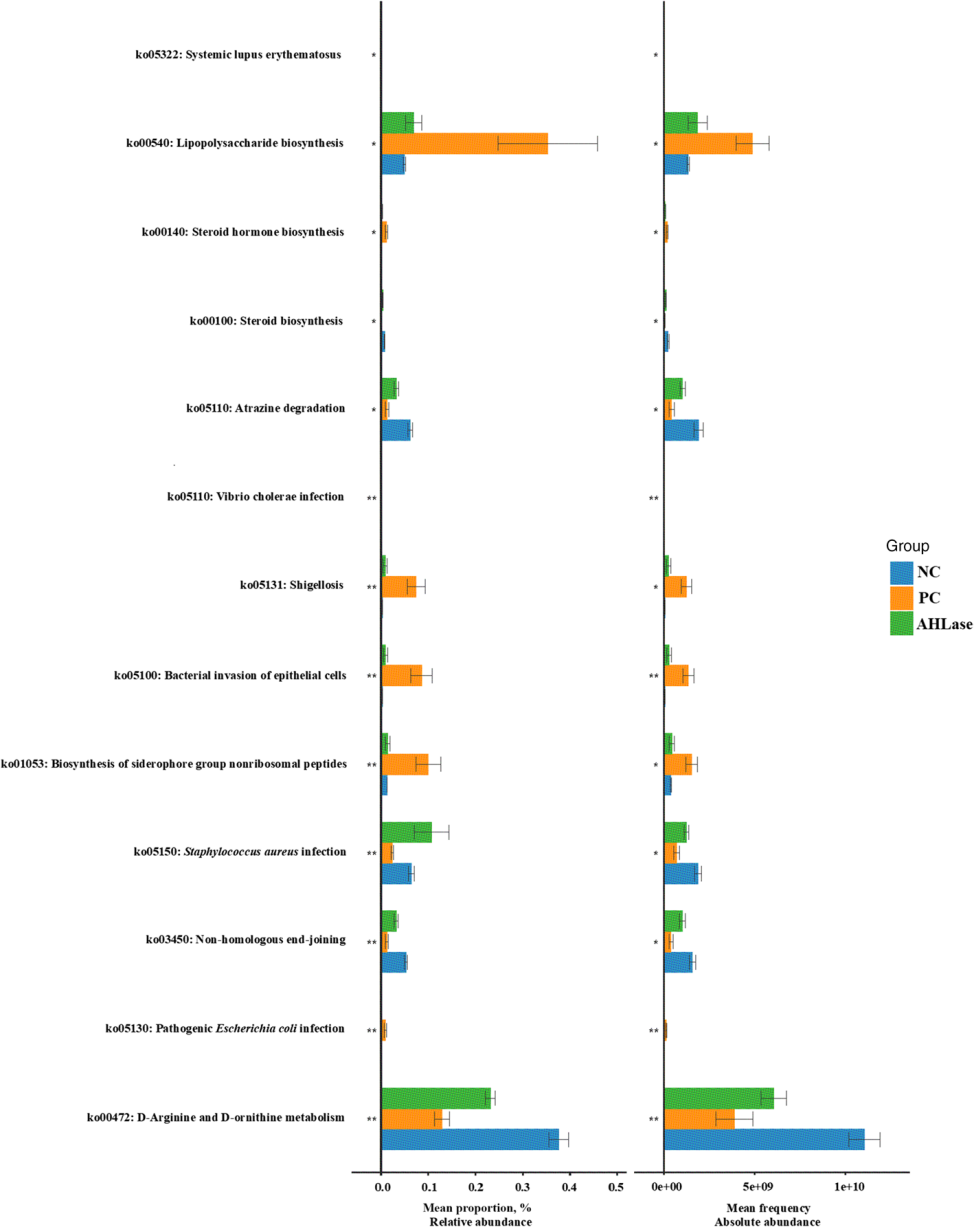
Functional prediction of gut microbiota of broilers on d 10 based on KEGG pathways. Differences among groups were identified by Kruskal-Wallis analysis. NC, negative control (broilers were free of challenge); PC, positive control (broilers were challenged with *S.* Typhimurium); AHLase, PC broilers supplemented with 10 U/g AHLase

Since there were obvious differences in gut microbial composition among groups, we then examined the putative functions of gut metagenome using the PICRUSt. According to both the RA and AA of functional genes implicated in KEGG pathways (Fig. 8), there were differences (*P* < 0.05) in the enrichments of certain pathways of gut microbiota among groups. Thereinto, the enrichments of bacterial pathogenicity-associated pathways such as lipopolysaccharide biosynthesis (ko00540), shigellosis (ko05131), bacterial invasion of epithelial cells (ko05100), biosynthesis of siderophore group nonribosomal peptides (ko01053) and pathogenic *Escherichia coli* infection (ko05130) in AHLase group were lower (*P* < 0.05) than PC group but comparable to NC group. Comparatively, the enrichments of the pathways of non-homologous end joining (ko03450) along with *D*-arginine and *D*-ornithine metabolism (ko00472) in AHLase group were elevated (*P* < 0.05) versus PC group and close to NC group.

#### Correlations of gut microbiota with intestinal parameters

**Fig. 9:**
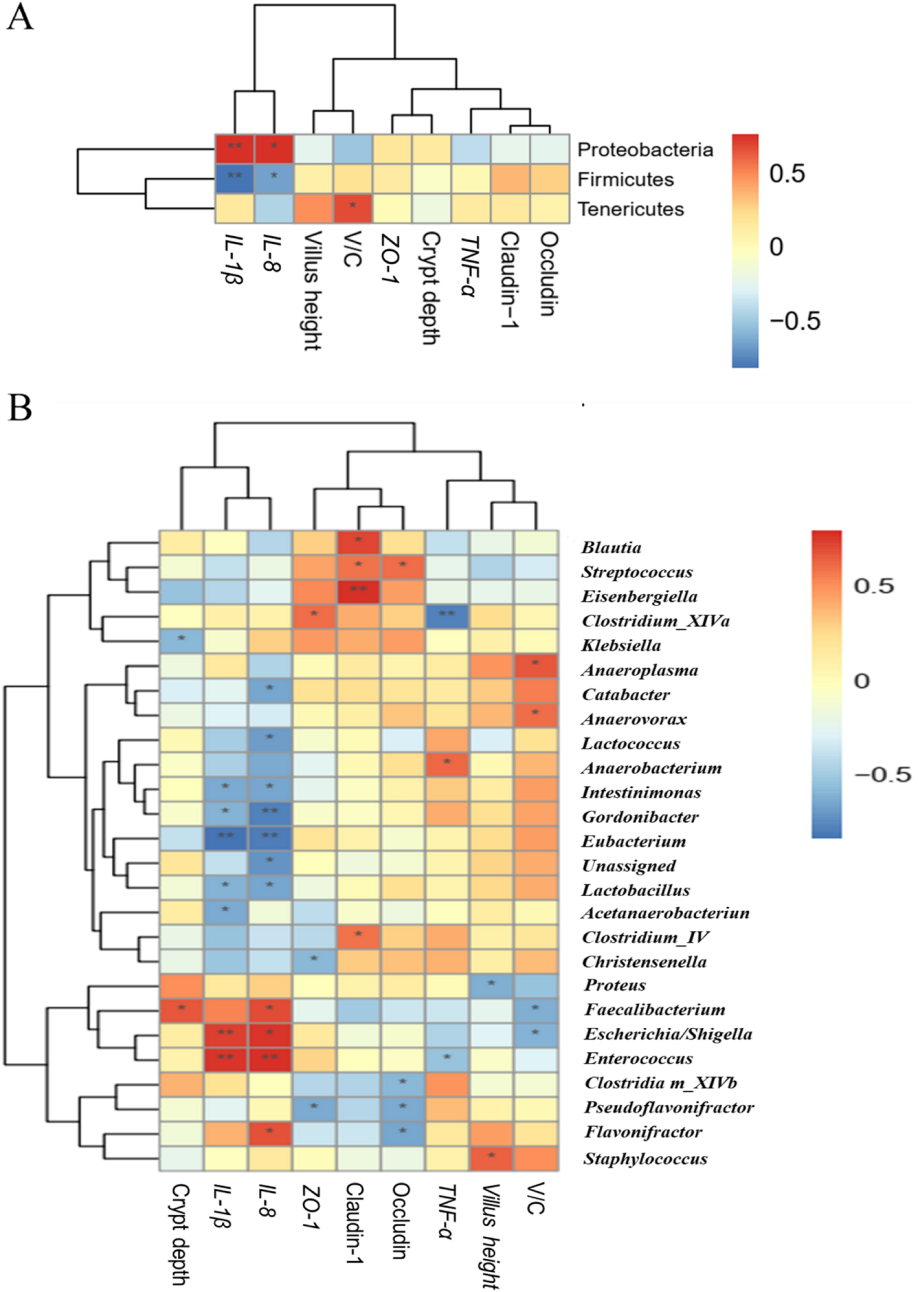
Correlation analysis between gut microbiota (**A**, at phyum level; **B**, at genus level) and intestinal parameters in broilers on d 10. *IL*, interleukin; *ZO*, zonula occludens; *TNF*, tumor necrosis factor; V/C, villus height to crypt depth ratio. The red and blue panes represent positive and negative correlations, respectively. Color intensity means the Spearman’s *r*-value of correlations in each panel. The asterisks indicate significant correlations (^∗^*P* < 0.05; ^∗∗^*P* < 0.01). NC, negative control (broilers were free of challenge); PC, positive control (broilers were challenged with *S.* Typhimurium); AHLase, PC broilers supplemented with 10 U/g AHLase

Certain potentially harmful bacteria such as Proteobacteria, Gammaproteobacteria, Enterobacteriales (Enterobacteriaceae), Enterococcaceae (*Enterococcus*) and *Escherichia*/*Shigella* had positive correlations (*P* < 0.05) with ileal *IL-1β* and *IL-8* expression (Fig. 9 and Fig. S3), while Firmicutes, Lactobacillaceae, Eubacteriaceae (*Eubacterium*), *Intestinimonas*, *Gordonibacter* and *Lactobacillus* were negatively correlated (*P* < 0.05) with ileal *IL-1β* and *IL-8* expression. Besides, Clostridia, Bacillales, Lactobacillaceae, Clostridiales_Incertae_Sedis_XIII, Lachnospiraceae and Eubacteriaceae showed negative correlations (*P* < 0.05) with ileal *IL-8* or *IL-1β* expression. Intriguingly, certain bacterial members such as Bacilli, Mollicutes, Anaeroplasmatales and Clostridiales_Incertae_Sedis_XIII elicited positive correlations (*P* < 0.05) with ileal V/C, whereas the contrasting pattern was found for *Escherichia/Shigella*.

#### Correlations of gut microbiota with serum DAO activity and growth performance

**Fig. 10:**
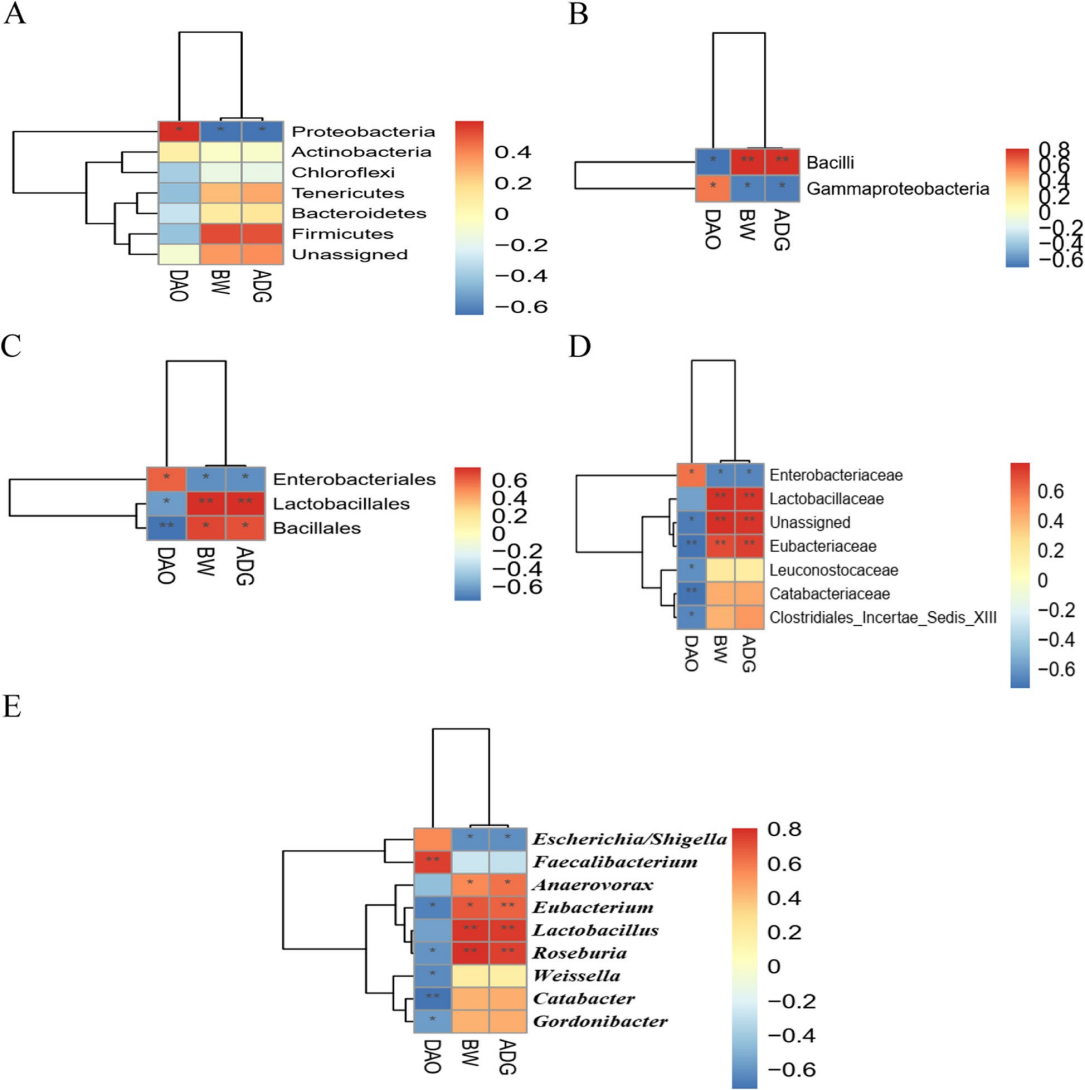
Correlation analysis of gut microbiota (**A**, at phyum level; **B**, at class level; **C**, at order level; **D**, at family level; **E**, at genus level) with serum diamine oxidase (DAO) activity and growth performance (BW, body weight; ADG, average daily gain) in broilers on d 10. The red and blue panes represent positive and negative correlations, respectively. Color intensity indicates the Spearman’s *r*-value of correlations in each panel. The asterisks indicate significant correlations (^∗^*P* < 0.05; ^∗∗^*P* < 0.01). NC, negative control (broilers were free of challenge); PC, positive control (broilers were challenged with *S.* Typhimurium); AHLase, PC broilers supplemented with 10 U/g AHLase

As illustrated in Fig. 10, there were positive correlations (*P* < 0.05) of the abundances of Proteobacteria, Gammaproteobacteria, Enterobacteriales and Enterobacteriaceae with serum DAO activity, however, these bacteria together with *Escherichia*/*Shigella* were negatively correlated (*P* < 0.05) with growth performance (BW and ADG). Notably, certain bacteria such as Bacilli (Bacillales), Lactobacillales, Eubacteriaceae (*Eubacterium*), Leuconostocaceae, Incertae Sedis XIII, *Weissella* and *Gordonibacter* were negative correlated (*P* < 0.05) with serum DAO activity. Comparatively, Bacilli (Bacillales), Lactobacillales (Lactobacillaceae, *Lactobacillus*) and Eubacteriaceae (*Eubacterium*) showed positive correlations (*P* < 0.05) with growth performance (BW and ADG).

## Discussion

Consistent with previous studies [1, 2], we observed the adverse impacts of *S.* Typhimurium challenge on broiler growth performance, as exhibited by the reductions of BW on both d 10 and 14 together with ADG during d 1–10 and d 1–14. Treatment with AHLase or AHL-degrading probiotics was reported to improve growth performance of aquatic animals [16, 21]. Likewise, the present study confirmed an alleviation of *S.* Typhimurium-induced reductions of BW and ADG in response to AHLase addition especially at a dosage of 10 U/g. This could be due to the ability of AHLase to degrade AHL and inhibit AHL-dependent QS, subsequently weakening the infectivity of *S.* Typhimurium and mitigating the resultant intestinal damages of host [8–11]. Based on the above findings, the dosage of 10 U/g was selected for further investigation. It could be natural that AHLase-induced promotion of growth performance in challenged birds was associated with the observed ability of it to attenuate intestinal disruption, because intestinal health has a close connection with animal growth performance [1, 2].

The relative weight of visceral organs is a common parameter reflecting bacterial infection status and immune function of chickens [22]. In this study, we found an increased relative weight of spleen and liver in challenged broilers, which might be ascribed to that intestinal *Salmonella* invaded into spleen and liver via destruction of intestinal integrity and subsequently caused compensatory hypertrophy of these organs [23]. In support of this view, we indeed detected down-regulations of intestinal intraepithelial tight junction (TJ) proteins (Claudin-1 and Occludin) in response to *S.* Typhimurium challenge. However, AHLase addition failed to moderate *S.* Typhimurium-induced increase in the relative weight of liver and spleen of broilers, which coincided with the observation that AHLase addition did not affect the expression of intestinal TJ proteins in challenged birds. This result implied that dietary AHLase elicited marginal benefit on the immune irritation of broiler visceral organs induced by *S.* Typhimurium challenge.

As a highly active intracellular enzyme secreted by intestinal epithelia, DAO can be released into blood circulation when there is a destruction of intestinal barrier [24]. Thus, blood DAO level acts as an indicator for monitoring the severity of intestinal disruption in broilers [2, 25]. It was reported that *Salmonella*-induced intestinal disruption was accompanied by an increase in serum DAO activity in broilers [2]. Likewise, we herein verified an increase in serum DAO activity in *S.* Typhimurium-challenged birds on d 10 instead of d 14, demonstrating that *S.* Typhimurium might prefer to destruct intestinal barrier of broilers at an early stage of infection. Supplemental AHLase attenuated the increase in serum DAO activity of challenged birds on d 10, highlighting a role of AHLase in protecting intestinal barrier of broilers against *S.* Typhimurium invasion.

Intestinal mucosa is an important component of intestinal physical barrier that separates gut lumen from the protected internal milieu of poultry [26, 27]. Ameliorations of intestinal mucosal morphology such as reduced CD along with increased VH and V/C can benefit intestinal barrier apart from the absorption capacity [26, 27]. In concert with previous studies [1, 2], we found that *S.* Typhimurium challenge impaired intestinal morphology of broilers on d 10, which could correspond to the synchronous increase in serum DAO activity in challenged birds. Previously, a study in zebrafish revealed that AHLase addition improved intestinal morphology, as characterized by increases in VH and goblet cell density [16]. In this study, we noted that AHLase addition counteracted the compromise of intestinal morphology including the elevated CD and reduced V/C of jejunum and ileum in challenged birds. This result coincided with the observed mitigatory effect of AHLase addition on the increased serum DAO activity in challenged birds.

Another important constituents of intestinal physical barrier are intraepithelial tight junctions (TJ) that comprise of multiple structurally unique proteins [28]. TJ form a paracellular permeability barrier and maintain intestinal integrity, functioning as a fence restraining translocation of antigens from gut lumen [28]. It was reported that *S.* Typhimurium invasion could disturb intestinal TJ in broilers by inhibiting the expression of certain TJ proteins [27]. Analogously, we recorded that *S.* Typhimurium-challenged birds had a reduced expression of ileal claudin-1 and occludin that are known as the core transmembrane proteins to maintain TJ structure. Nevertheless, AHLase addition to *S.* Typhimurium-challenged birds did not reverse the reduced expression of ileal TJ proteins, hinting an inability of AHLase to abolish the detriment of *S.* Typhimurium to intestinal integrity of broilers.

Inflammatory cytokines are well-established as regulators of intestinal integrity and mucosal morphology [29]. It is increasingly recognized that inflammatory cytokine-mediated inflammation exerts momentous pathological roles in contributing to *Salmonella*-induced intestinal barrier dysfunction in animals [2, 23]. However, there can be varying responses (either increase or reduction) of intestinal inflammatory cytokines expression in broilers to *S.* Typhimurium challenge that likely depends on the time-points post infection and sampling location [30]. In this study, we found a time-dependent alteration of ileal *IL-1β*, *IL-8* and *TNF-α* expression responding to *S.* Typhimurium challenge, confirming a complicated impact of *S.* Typhimurium on the expression profile of intestinal inflammatory cytokines presumptively due to the intricate immune feedbacks of host [31]. It is known that AHLase can weaken the pathogenicity and infectivity of certain Gram-negative pathogens by restraining their physiological activities such as biofilm formation and toxin secretion [10–15]. Specially, it was found to reverse *Pseudomonas aeruginosa*-induced acute pneumonia by alleviating inflammatory cytokines expression in mice [12]. In this study, AHLase addition counteracted *S.* Typhimurium*-*induced increase in ileal *IL-1β* and *IL-8* expression of broilers on d 10, validating a mitigatory effect of AHLase on intestinal inflammation in challenged birds. This could contribute to the observed improvement of intestinal barrier function in challenged birds following AHLase addition*.*

Gut microbiota can mediate *Salmonella*-induced inflammatory environment in gut [32], and has profound roles in determining intestinal health and growth performance of chickens especially those under infection condition [2, 33]. Consistent with previous studies [2, 33], we noted that *S.* Typhimurium challenge did not change α-diversity but distinctly altered β-diversity (similarity) of broiler gut microbiota, as visualized by both PCA, PCoA and NMDS plots. Nevertheless, this alteration was abolished by AHLase addition. Bacterial abundance analysis supported huge changes in gut microbial composition among groups. For example, *S.* Typhimurium challenge favored an increase in Proteobacteria at the expense of Firmicutes, whereas AHLase addition reversed this phenomenon. Proteobacteria is a microbial signature of gut microbiota dysbiosis and epithelial dysfunction because of its composition of abundant pathogens (e.g. *Salmonella* and *Shigella*) that can generate toxins resulting in intestinal inflammation and dysfunction [34, 35]. Inversely, Firmicutes includes many important beneficial bacteria (e.g. *Bacillus* and *Lactobacillus*), benefiting the ability of host to harvest energy from diet [36]. A number of studies have evidenced a linkage of increased Proteobacteria and decreased Firmicutes with intestinal inflammatory injuries and growth impairment in chickens [37, 38]. Similarly, we observed that Proteobacteria showed a positive correlation with ileal inflammatory cytokines (*IL-1β* and *IL-8*) expression and serum DAO activity, along with a negative correlation with BW and ADG, whereas Firmicutes was negatively correlated with ileal *IL-1β* and *IL-8* expression. Within Firmicutes, several potentially beneficial bacteria including Bacilli, Bacillales, Lactobacillales, Lactobacteriaceae, Clostridiales_Incertae_Sedis_XIII, Eubacteriaceae, *Eubacterium, Anaerobacterium* and *Intestinimonas* were identified to distinguish gut microbiota among groups. Thereinto, Bacilli (Bacillales) serve as natural guards in host gut against bacterial invasion and intestinal pathologies [39]. Lactobacillales including Lactobacteriaceae and *Lactobacillus* are the typical beneficial bacteria and major sources of probiotics, exerting crucial roles in prompting intestinal health and growth of animals by restricting gut inflammation and barrier dysfunction [40, 41]. Clostridiales_Incertae_Sedis_XIII may also promote intestinal homeostasis due to its implication in mediating the beneficial effects of prebiotics on intestinal homeostasis and growth performance of animals [42]. Eubacteriaceae including *Eubacterium* are regarded as important regulators of bile acid metabolism and producers of short-chain fatty acids possibly due to their capacity to decompose polysaccharides in plant-based diets, thereby favoring animal growth and intestinal health [43, 44]. *Anaerobacterium* and *Intestinimonas* represent momentous bacterial groups linked with production of butyrate [45, 46], which functions as a key nutrient component for intestinal epithelia supporting anti-inflammation and damage recovery of gut [47], thus benefiting protection of intestinal health and growth performance of broilers [48]. In this study, AHLase addition enriched Bacilli, Bacillales, Lactobacillales, Lactobacteriaceae, Clostridiales_Incertae_Sedis_XIII, Eubacteriaceae, *Lactobacillus*, *Eubacterium*, *Anaerobacterium* and *Intestinimonas* within Firmicutes in broiler gut. More notably, most of the these bacteria were negatively correlated with ileal inflammatory cytokine (*IL-1β* or *IL-8*) expression and serum DAO activity, and positively correlated with BW and ADG. These results supported an idea that the elevations in these beneficial bacteria following AHLase addition were probably associated with the simultaneous alleviation of *S.* Typhimurium-induced intestinal disruption and growth retardation of broilers.

In addition to enriching the beneficial bacteria, AHLase addition also lowered several potentially harmful bacteria such as Enterococcaceae, *Enterococcus* and *E. cecorum* within Firmicutes, as well as Gammaproteobacteria, Enterobacteriales, Enterobacteriaceae and *Escherichia*/*Shigella* within Proteobacteria in broiler gut. Thereinto, increased Enterococcaceae and *Enterococcus* are often companied by pathogenicity and antibiotic resistance coupled with a potential induction of gut microbiota dysbiosis and pathological injury [49, 50]. Among *Enterococcus*, *E. cecorum* is the most frequently occurring enterococcal species in poultry gut and serve as an important opportunistic pathogen for chickens [51]. As a critical order of Gammaproteobacteria, Enterobacteriales is comprised of numerous harmful families especially the Enterobacteraceae, which includes several typical pathogenic or opportunistic genera such as pathogenic *Escherichia*, *Shigella*, *Salmonella* and *Klebsiella* [52]. Consequently, we speculated that the loss of Gammaproteobacteria including Enterobacteriales and Enterobacteriaceae following AHLase addition was primarily owe to the decrease of *Escherichia*/*Shigella*. Similarly, the loss of Enterococcaceae in AHLase group was likely responsible by the reduction of *Enterococcus* particularly the *E. cecorum*. Previously, Gammaproteobacteria, Enterobacteriales, Enterobacteriaceae and *Escherichia*/*Shigella* were validated to actively trigger gut microbota dysbiosis with inflammatory disruption of host intestine [53–55], subsequently connecting with an impairment of broiler growth performance [56, 57]. In this study, AHLase addition decreased certain potentially harmful bacteria (e.g. Proteobacteria, Gammaproteobacteria, Enterobacteriales, Enterobacteriaceae, Enterococcaceae, *Escherichia*/*Shigella*, *Enterococcus* and *E. cecorum*), furthermore, most of these bacteria were positively correlated with ileal inflammatory cytokines (*IL-1β* and *IL-8*) expression and serum DAO activity, and negatively correlated with BW and ADG. These findings suggested that the reductions in these harmful bacteria together with increases in the above-mentioned beneficial bacteria could contribute to the observed reversing effect of AHLase on intestinal disruption and growth retardation in *S.* Typhimurium-challenged broilers. Similarly, previous studies in aquatic animals revealed that supplemental AHLase or AHL-degrading probiotics promoted the growth and intestinal health by improving gut microbial composition, characterized by the increases and decreases in certain beneficial and harmful bacteria, respectively [16, 21].

In an attempt to better understand the cause of the protective effects of AHLase for broilers, we focused on the comparison of functional pathways of gut microbiota among groups. The results showed that AHLase addition counteracted the *S.* Typhimurium-induced upregulations of certain specialized pathways (e.g. lipopolysaccharide biosynthesis, shigellosis, bacterial invasion of epithelial cells, pathogenic *Escherichia coli* infection and biosynthesis of siderophore group nonribosomal peptides) in broiler gut microbiota. Lipopolysaccharide, also known as endotoxin, is a key virulence factor of Gram-negative pathogens with a high ability to irritate host inflammatory responses, thus triggering intestinal damages and compromised growth performance in broilers [58]. Shigellosis is generally caused by *Shigella* and shiga-toxin producing *E. coli,* which strongly destroys intestinal structure and functions by inhibiting protein synthesis and facititating cell apoptosis of epithelial cells [59, 60]. This could be supported by the synchronous changes in the pathways of bacterial invasion of epithelial cells and pathogenic *Escherichia coli* infection of gut microbiota. Siderophore, a small-molecule iron ion-chelating agent secreted by certain bacteria, can be sensed by specific outer membrane receptors and transported into cytoplasm to provide iron for bacteria, thereby assisting with bacterial infection [61]. It was demonstrated that the downregulated pathway of biosynthesis of siderophore group nonribosomal peptides of gut microbiota might prompt animals to maintain intestinal homeostasis during gut inflammation [62]. To sum up, the normalization of pathogenicity-related microbial pathways (lipopolysaccharide biosynthesis, shigellosis, bacterial invasion of epithelial cells, pathogenic *Escherichia coli* infection and biosynthesis of siderophore group nonribosomal peptides) could also conduce to the observed protective effects of AHLase addition against *S.* Typhimurium-induced intestinal disruption and subsequently favored normalization of growth performance in broilers.

## Conclusions

Supplemental AHLase at a dosage of 10 U/g attenuated *S.* Typhimurium-induced growth retardation and intestinal disruption by alleviating intestinal inflammation and barrier dysfunction in broilers. This could be at least partially associated with its capacity to mitigate *S.* Typhimurium-induced gut microbiota dysbiosis, as evidenced by the increases in several beneficial bacteria (e.g. *Lactobacillus* and *Eubacterium*) and the reductions of certain harmful bacteria (e.g. *Escherichia*/*Shigella* and *Enterococcus*), together with the downregulations of bacterial pathogenicity-related pathways (e.g. lipopolysaccharide biosynthesis). Our findings verified the roles of gut microbiota in mediating the protective actions of AHLase (a quorum quenching enzyme) for growth and intestinal health of broilers, thus providing a new insight into the control of *Salmonella* infection in animals.

## Supplementary Information

**Additional file 1: Table S1.** Sequences for real-time PCR primers. **Fig. S1.** Alpha-diversity analysis of gut microbiota among groups. **Fig. S2.** Proportions (%) of bacterial members in gut microbiota among groups. **Fig. S3.** Correlation analysis between gut microbiota (**A**, at class level; **B**, at order level; **C**, at family level) and intestinal parameters in broilers on d 10.

## Data Availability

All data in this study are available from the corresponding author. The datasets supporting the conclusions of this study are included in the main text and supplemental materials of this manuscript.

## Abbreviations

AA: Absolute abundance
ADG: Average daily gain
AHL: N-Acyl-homoserine lactone
AHLase: N-Acyl homoserine lactonase
BW: Body weight
CD: Crypt depth
DAO: Diamine oxidase
LDA: Linear discriminant analysis
LefSe: LDA combined effect size measurements
IL: Interlukin
NC: Negative control
NMDS: Non-metric multidimensional scaling
PC: Positive control
PCA: Principal component analysis
PcoA: Principal coordinates analysis
PICRUSt: Phylogenetic Investigation of Communities by Reconstruction of Unobserved State
QS: Quorum sensing
RA: Relative abundance
TNF: Tumor necrosis factor
VH: Villus height
V/C: Villus height to crypt depth
ZO: Zonula occluden
