# Supplementary material for: Supplemental N-acyl homoserine lactonase alleviates intestinal disruption and improves gut microbiota in broilers challenged by Salmonella Typhimurium
Source: J Anim Sci Biotechnol. 2023 Jan 9;14:7. doi: 10.1186/s40104-022-00801-4 (PMC9827655; doi:10.1186/s40104-022-00801-4)
Supplement: Supplementary file 1 — Additional file 1: Table S1. Sequences for real-time PCR primers. Fig. S1. Alpha-diversity analysis of gut microbiota among groups. Fig. S2. Proportions (%) of bacterial members in gut microbiota among groups. Fig. S3. Correlation analysis between gut microbiota (A, at class level; B, at order level; C, at family level) and intestinal parameters in broilers on d 10. [file 40104_2022_801_MOESM1_ESM.docx]

**Table S1** Sequences for real-time PCR primers

| Genes^1^ |  | Primer sequence^2^ (5′-3′) | Accession no. |
| --- | --- | --- | --- |
| *GAPDH* |  | F: GGGCACGCCATCACTATCTT | NM_204305 |
|  |  | R: TCACAAACATGGGGGCATCA |  |
| *IL-1β* |  | F: TGCCTGCAGAAGAAGCCTCG | Y15006.1 |
|  |  | R: GACGGGCTCAAAAACCTCCT |  |
| *IL-8* |  | F: TTGGAAGCCACTTCAGTCAGAC | DQ393272.2 |
|  |  | R: GGAGCAGGAGGAATTACCAGTT |  |
| *TNF-α* |  | F: GAGCAGGGCTGACACGGAT | GU230788.1 |
|  |  | R: CAGGCACAAAAGAGCTGATGG |  |
| *ZO-1* |  | F: CTTCAGGTGTTTCTCTTCCTCCTC | XM_413773 |
|  |  | R: CTGTGGTTTCATGGCTGGATC |  |
| Occludin |  | F: TTCGTCATGCTCATCGCCTC | D21837.1 |
|  |  | R: TCCACGGTGCAGTAGTGGTA |  |
| Claudin-1 |  | F: CACTGCCACTCCCTGATGTT | AY750897.1 |
|  |  | R: ACCGGTGACAGACTGGTTTC |  |

^1^ *GAPDH*, reduced glyceraldehyde-phosphate dehydrogenase; *IL*, interlukin; *TNF*, tumor necrosis factor; *ZO-1*, zonula occluden 1.

^2^ F, forward; R, reverse.

| 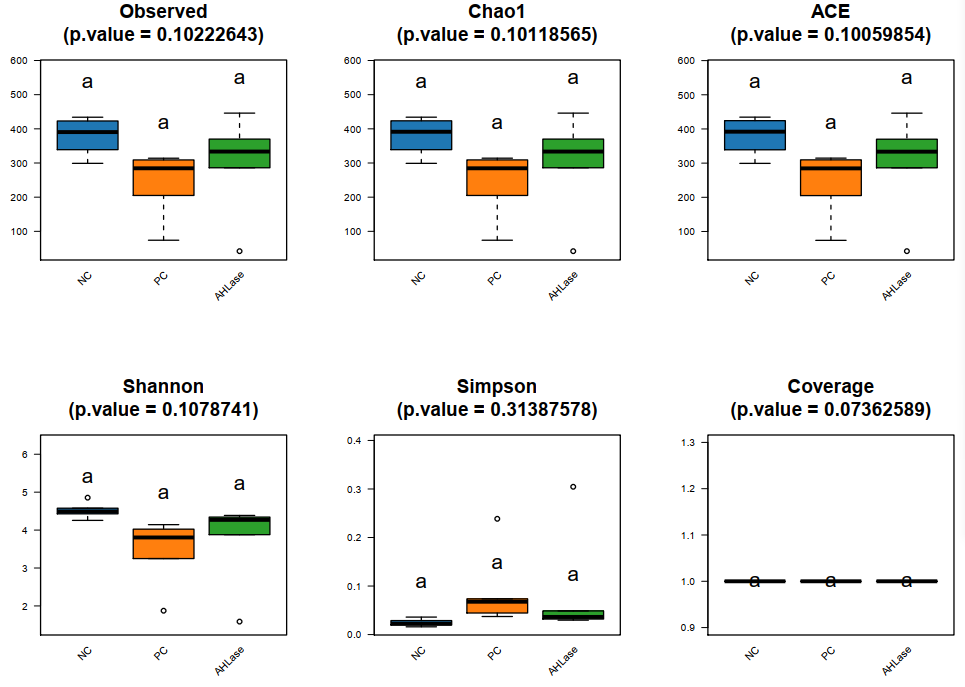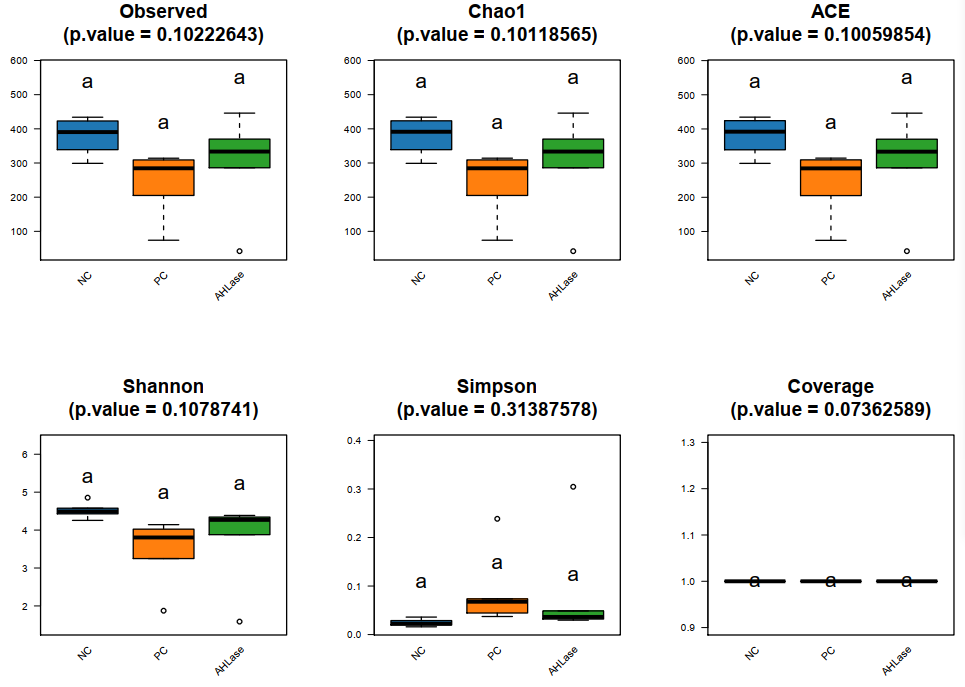 |
| --- |

**Fig. S1** Alpha-diversity analysis of gut microbiota among groups on d 10. NC, negative control (birds were free of challenge); PC, positive control (birds were challenged with *S.* Typhimurium from 7-9 d of age); AHLase, PC broilers supplemented with 10 U/g AHLase.

|  | **NC group** | **PC group** | **AHLase group** |
| --- | --- | --- | --- |
| Phylum | 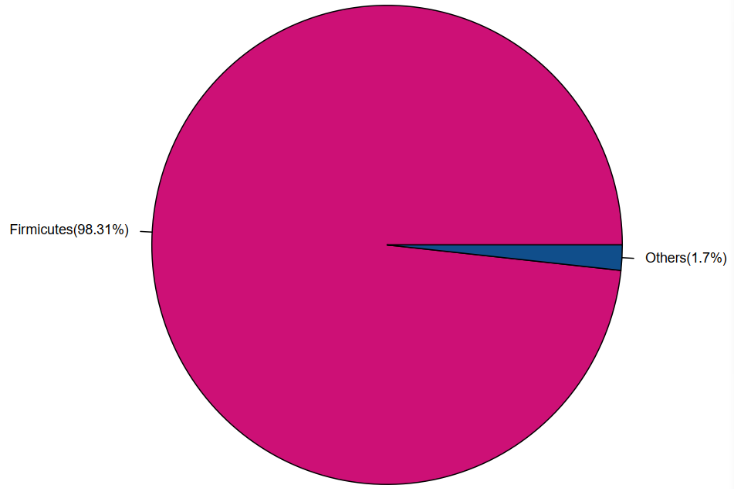 | 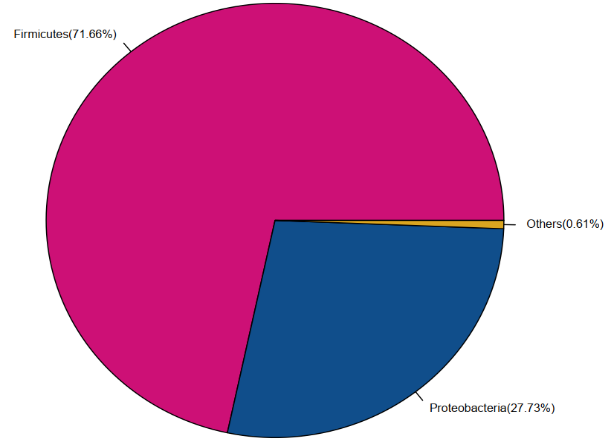 | 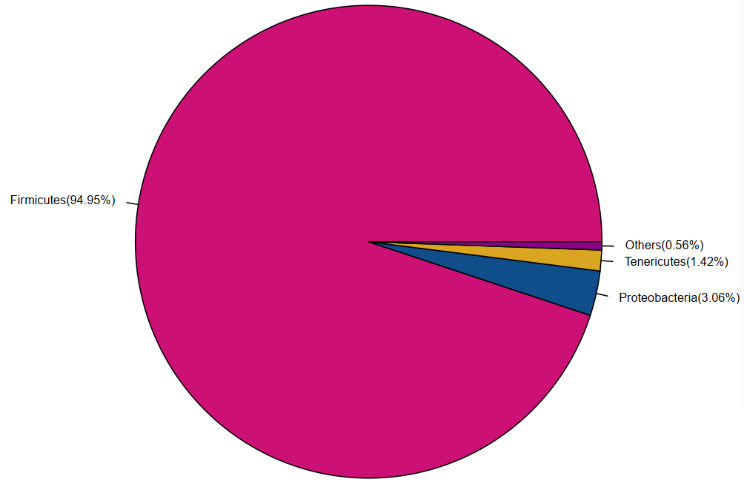 |
| Class | 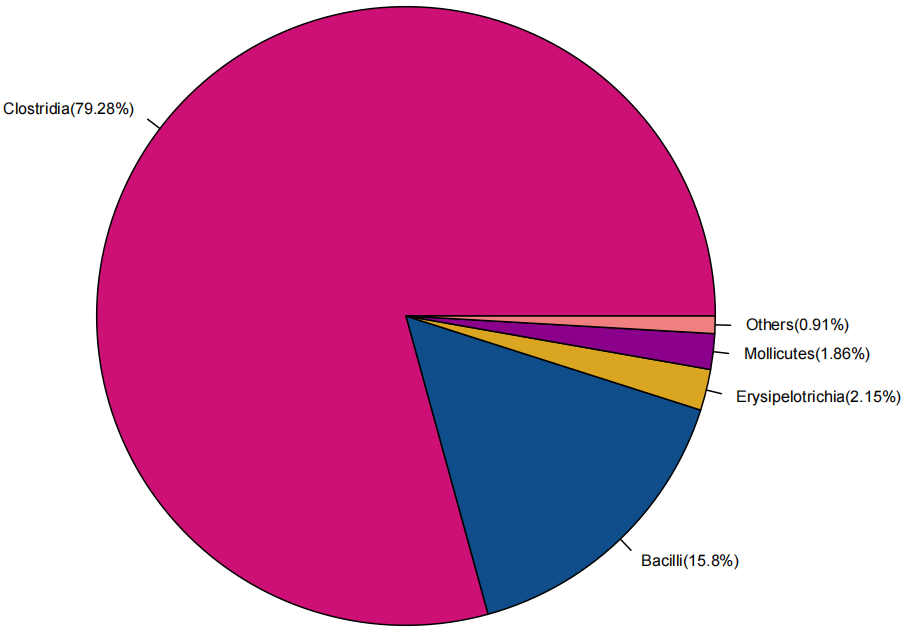 | 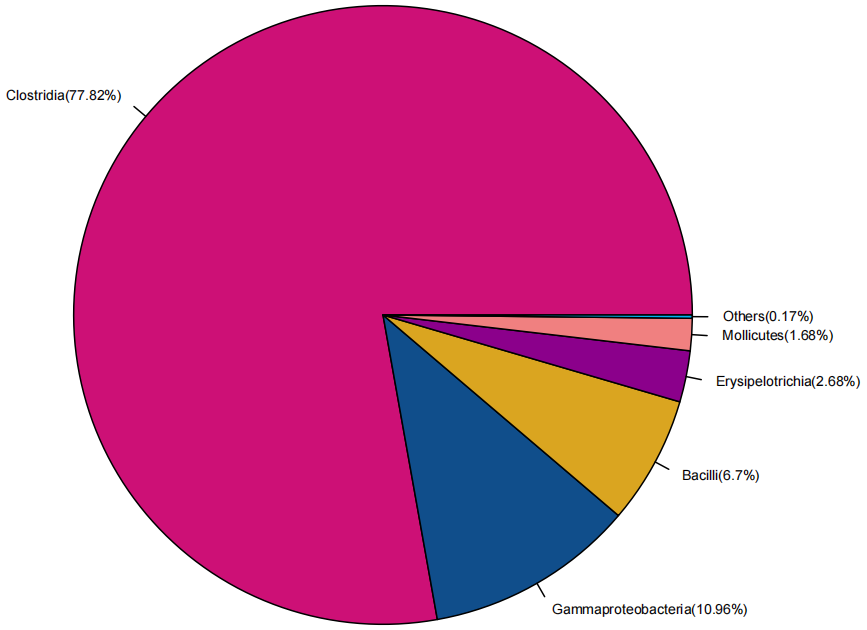 | 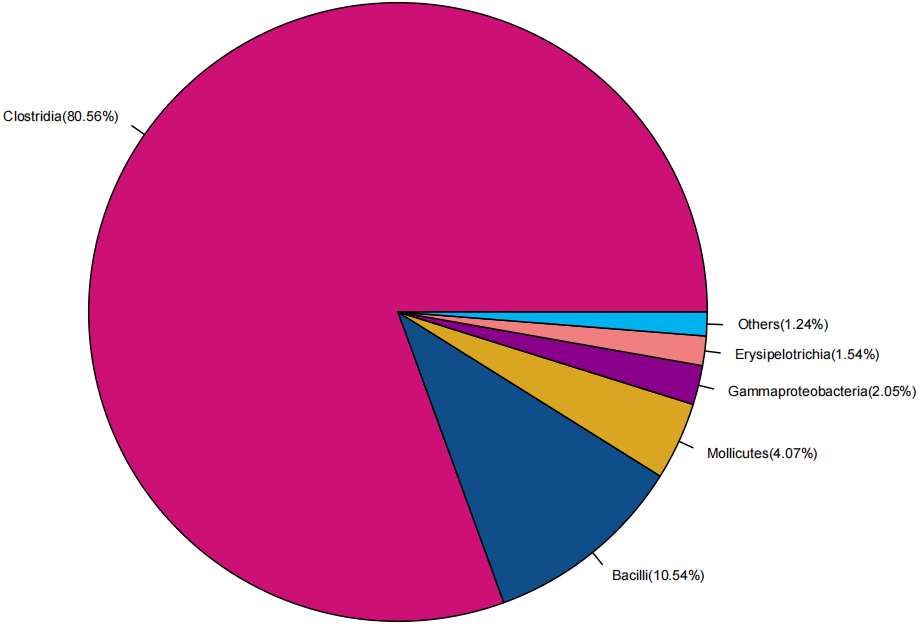 |
| Order |  |  |  |
|  | 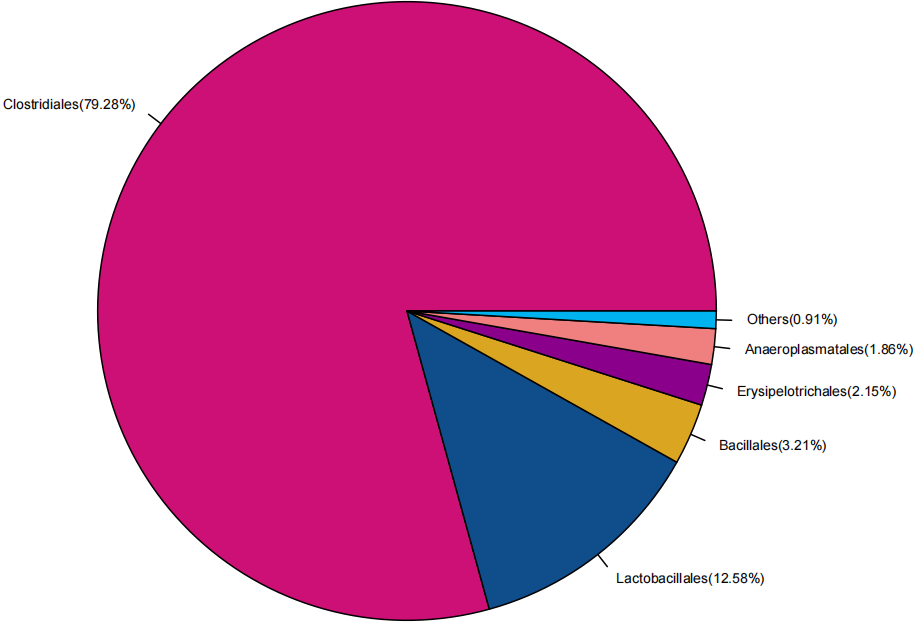 | 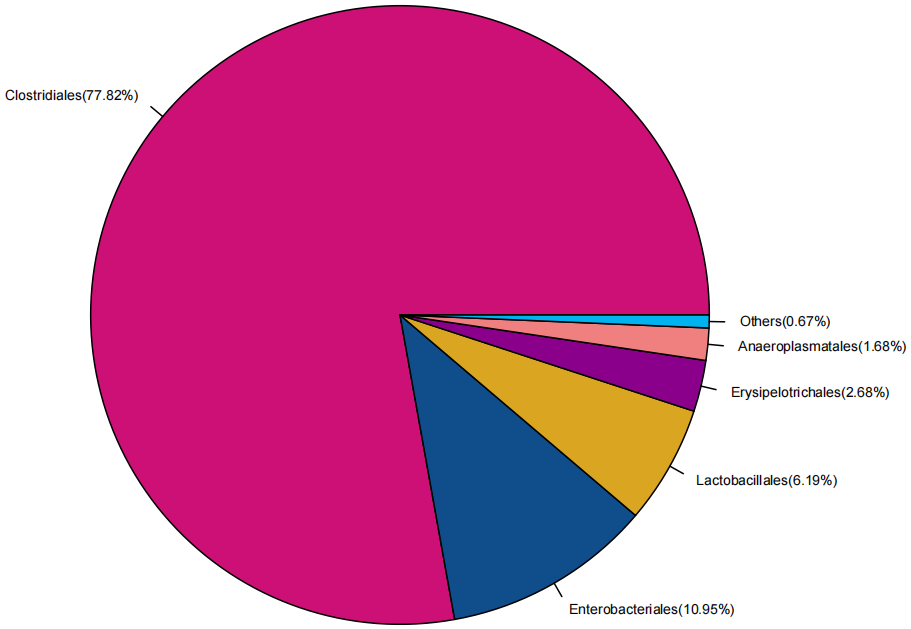 | 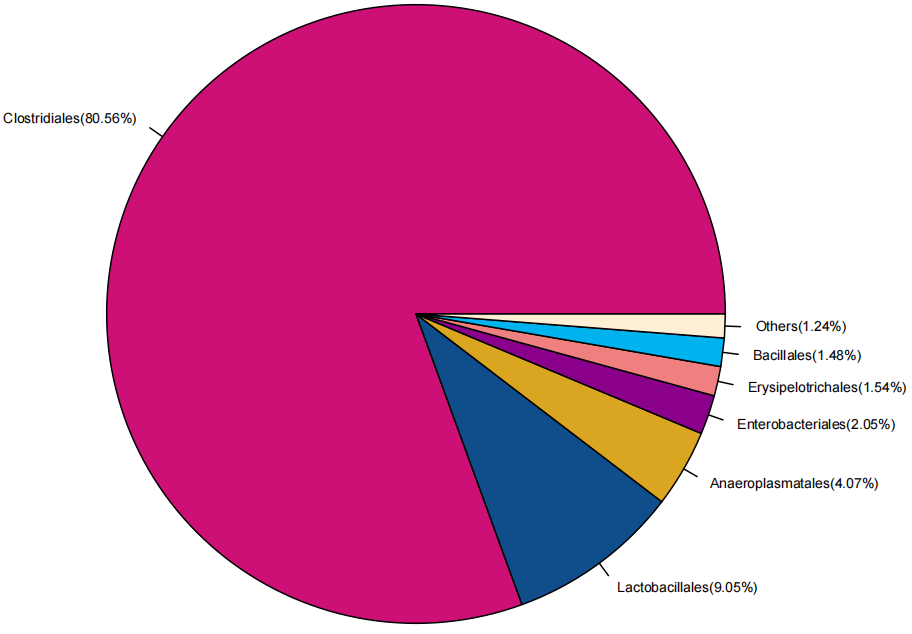 |
| Family | 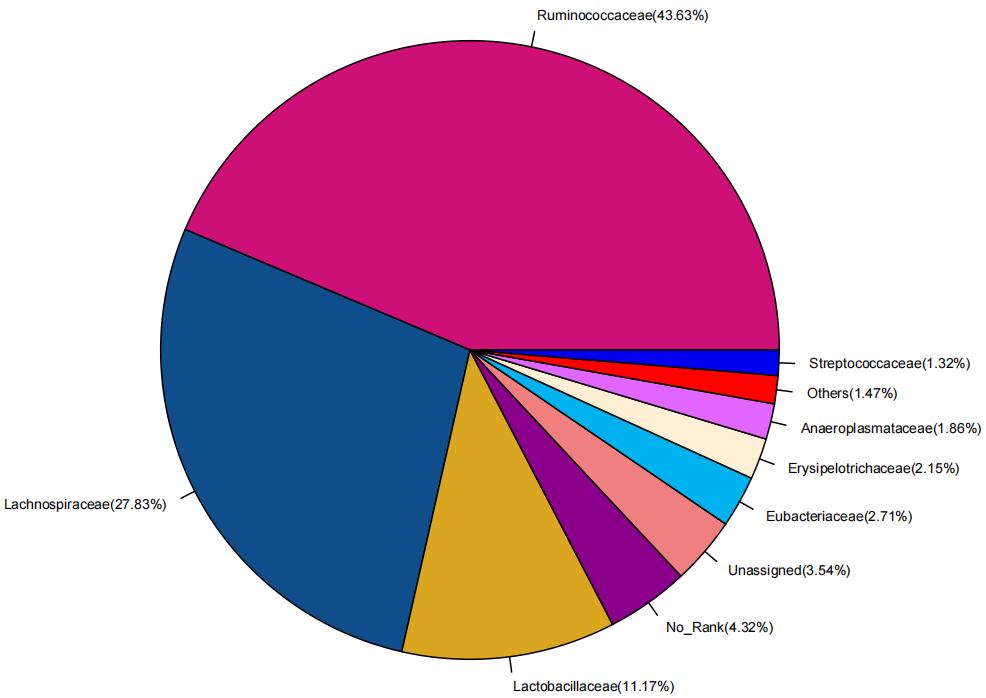 | 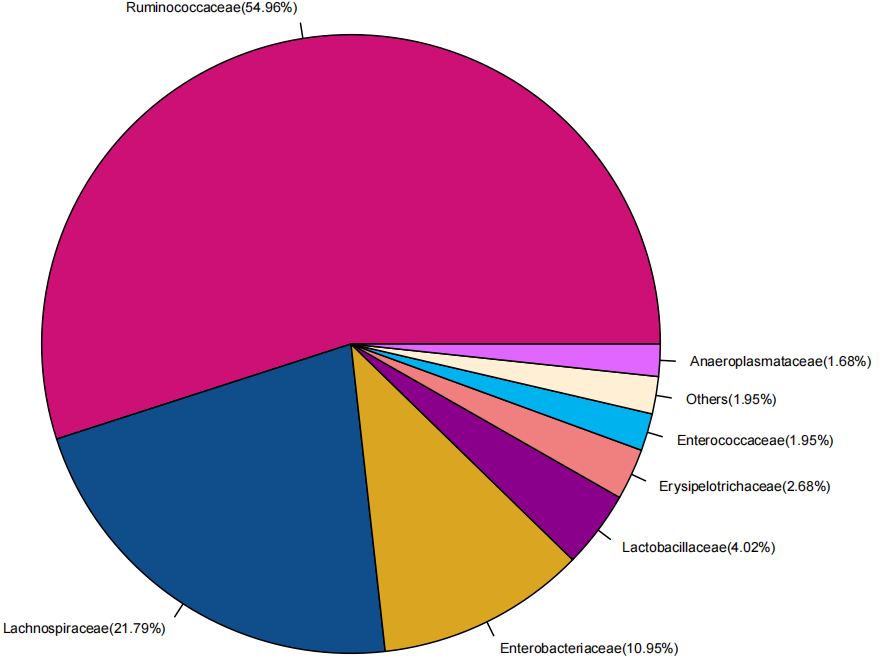 | 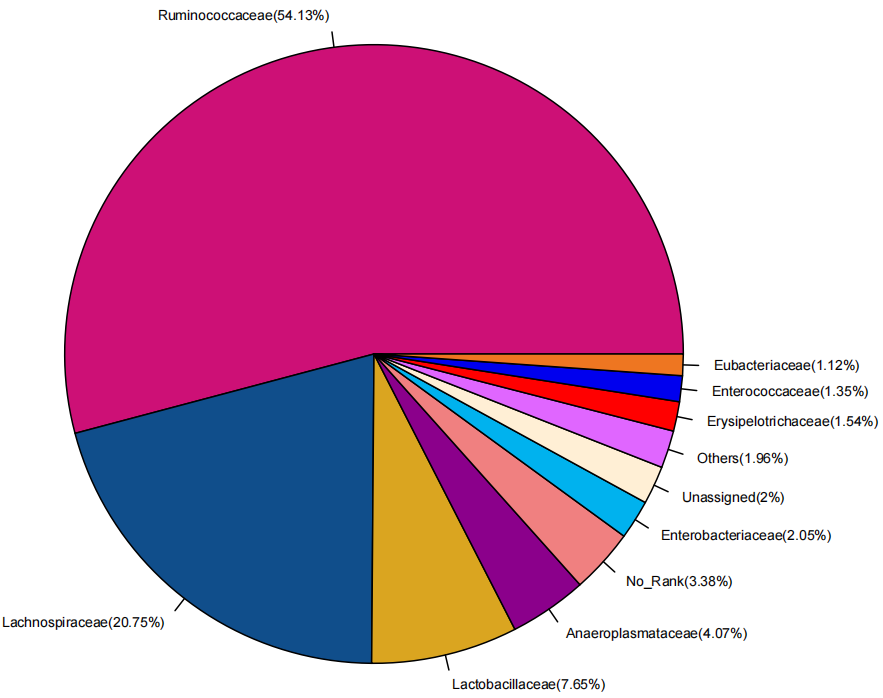 |
| Genus | 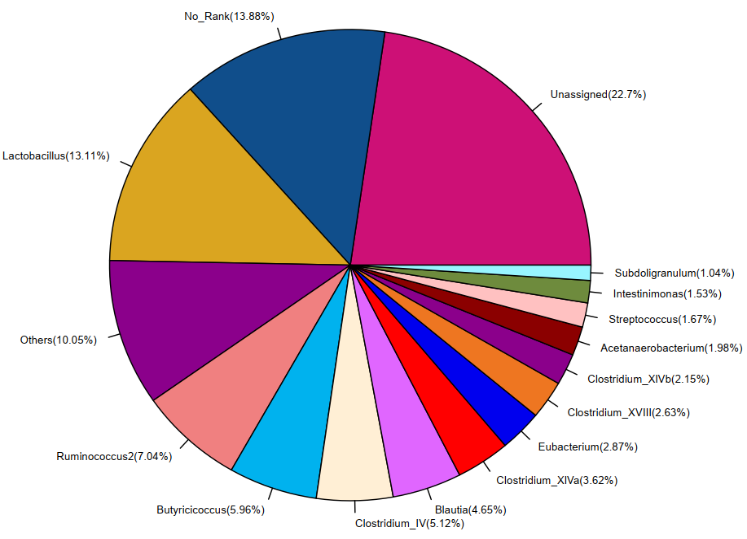 | 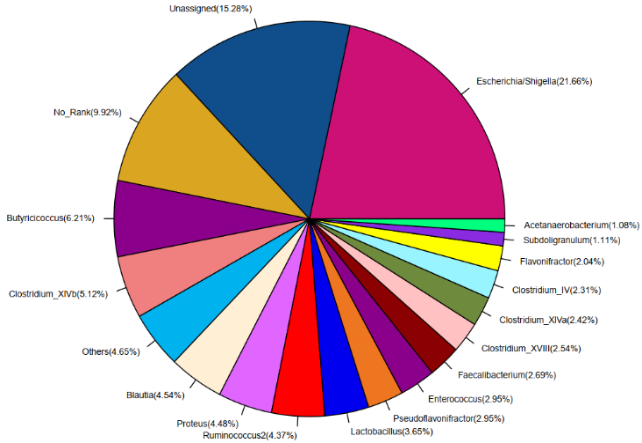 | 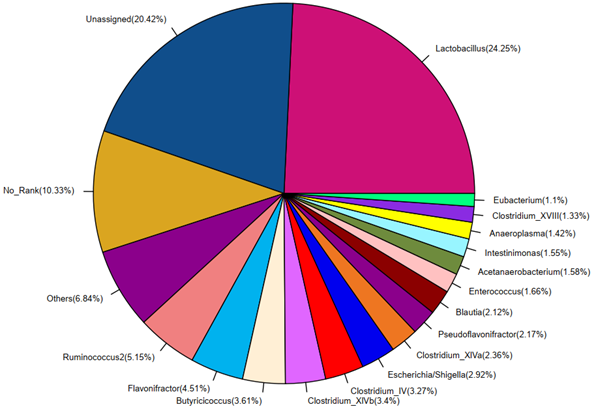 |

**Fig. S2** Proportions (%) of bacterial members in gut microbiota among groups on d 10. NC, negative control (birds were free of challenge); PC, positive control (birds were challenged with *S*. Typhimurium); AHLase, PC broilers supplemented with 10 U/g AHLase.

| A | B |
| --- | --- |
| 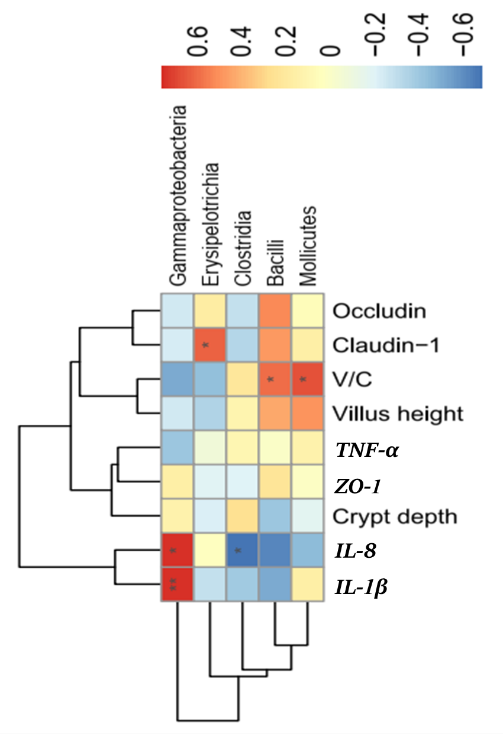 | 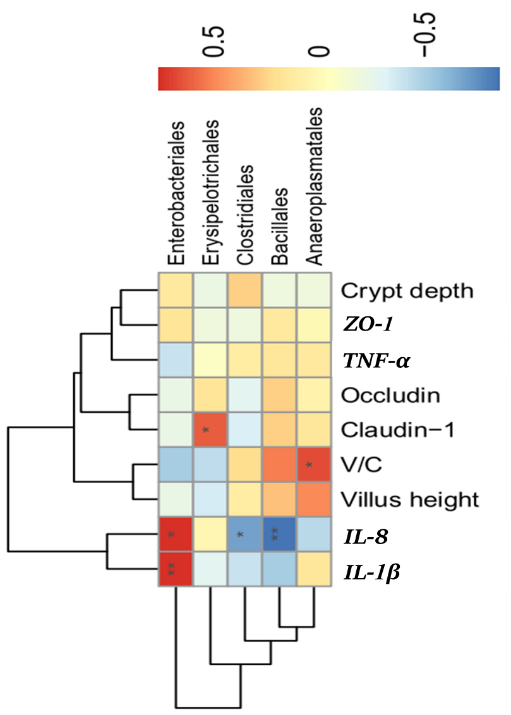 |
| C | |
| 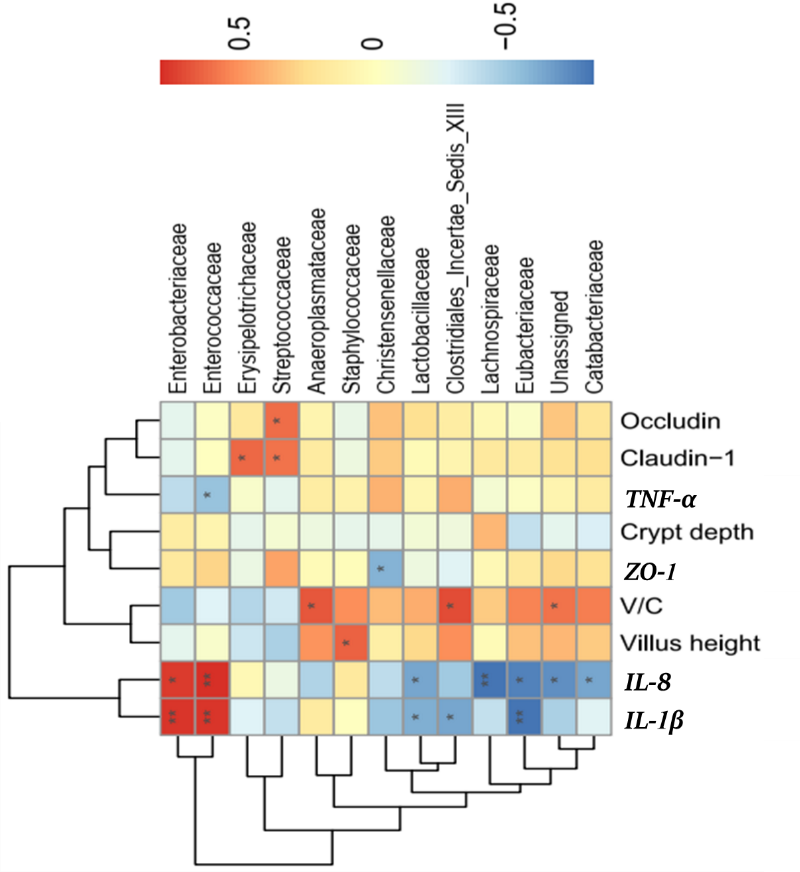 | |

**Fig. S3** Correlation analysis between gut microbiota (A, at class level; B, at order level; C, at family level) and intestinal parameters in broilers on d 10. *IL*, interleukin; *ZO*, zonula occludens; *TNF*, tumor necrosis factor; V/C, villus height to crypt depth ratio. The red and blue panes represent positive and negative correlations, respectively. Color intensity means the Spearman’s r-value of correlations in each panel. The asterisks indicate significant correlations (^∗^*P* < 0.05; ^∗∗^*P* < 0.01). NC, negative control (birds were free of challenge); PC, positive control (birds were challenged with *S.* Typhimurium); AHLase, PC broilers supplemented with 10 U/g AHLase.
